# Globally elevated levels of histone H3 lysine 9 trimethylation in early infancy are associated with poor growth trajectory in Bangladeshi children

**DOI:** 10.1186/s13148-023-01548-z

**Published:** 2023-08-11

**Authors:** Kristyna Kupkova, Savera J. Shetty, Marilyn G. Pray-Grant, Patrick A. Grant, Rashidul Haque, William A. Petri, David T. Auble

**Affiliations:** 1 Department of Biochemistry and Molecular Genetics, University of Virginia Health System, Charlottesville, VA 22908 USA; 2 Center for Public Health Genomics, University of Virginia Health System, Charlottesville, VA 22908 USA; 3 Department of Biomedical Science, Charles E. Schmidt College of Medicine, Florida Atlantic University, Boca Raton, FL 33431 USA; 4 Infectious Disease Division, International Centre for Diarrhoeal Disease Research, Dhaka, 1000 Bangladesh; 5 Division of Infectious Diseases and International Health, University of Virginia Health System, Charlottesville, VA 22908 USA

**Keywords:** Stunting, Childhood undernutrition, Epigenetics, Histone methylation, Heterochromatin, Endogenous retroviruses

## Abstract

**Background:**

Stunting is a global health problem affecting hundreds of millions of children worldwide and contributing to 45% of deaths in children under the age of five. Current therapeutic interventions have limited efficacy. Understanding the epigenetic changes underlying stunting will elucidate molecular mechanisms and likely lead to new therapies.

**Results:**

We profiled the repressive mark histone H3 lysine 9 trimethylation (H3K9me3) genome-wide in peripheral blood mononuclear cells (PBMCs) from 18-week-old infants (n = 15) and mothers (n = 14) enrolled in the PROVIDE study established in an urban slum in Bangladesh. We associated H3K9me3 levels within individual loci as well as genome-wide with anthropometric measurements and other biomarkers of stunting and performed functional annotation of differentially affected regions. Despite the relatively small number of samples from this vulnerable population, we observed globally elevated H3K9me3 levels were associated with poor linear growth between birth and one year of age. A large proportion of the differentially methylated genes code for proteins targeting viral mRNA and highly significant regions were enriched in transposon elements with potential regulatory roles in immune system activation and cytokine production. Maternal data show a similar trend with child’s anthropometry; however, these trends lack statistical significance to infer an intergenerational relationship.

**Conclusions:**

We speculate that high H3K9me3 levels may result in poor linear growth by repressing genes involved in immune system activation. Importantly, changes to H3K9me3 were detectable before the overt manifestation of stunting and therefore may be valuable as new biomarkers of stunting.

**Supplementary Information:**

The online version contains supplementary material available at 10.1186/s13148-023-01548-z.

## Background

Childhood stunting is a result of chronic undernutrition, and even with the available resources, stunting remains a global health problem affecting over 20% of children under the age of five worldwide [1]. Stunting is defined by a height-for-age z (HAZ) score below -2 and emerges within the first 1000 days post conception [1, 2]. Without treatment, stunting leads to a significant risk of mortality in children under the age of five and is associated with severe lifelong heath and socioeconomic problems. Common issues associated with stunting include immune system dysfunction [3, 4], cognitive impairment [5, 6], poverty, and paradoxically excessive weight gain later in life often resulting in obesity and with it diseases such as type 2 diabetes and cardiovascular diseases [7, 8]. While nutrient limitation plays a significant role in the development of stunting, it is not the sole driver. Many factors contribute to stunting, including socioeconomic factors [9–12], limited access to clean water and poor sanitation, which lead to an increased pathogen exposure and can result in enteric infections with or without diarrhea altering gut health, triggering chronic inflammation, and negatively affecting microbiome maturation [13–17]. Maternal health has been identified as another key factor in the development of stunting [16, 18]. Mothers of stunted children were often stunted themselves and evidence suggests they can pass this condition to neonates before birth [19] through mechanisms that are not well understood but that can impact subsequent generations [20, 21].

One of the global nutrition targets released in 2014 is to achieve a 40% reduction in stunting in children under five by 2025 [22]. While there has been a decline in the rate of stunting since then, the current interventions are not effective enough to reach this goal. Multiple studies have conducted metabolomic or metagenomic profiling in association with child growth to identify key metabolites for new nutritional therapies [13, 23–26]. In our approach, we aim to understand the detailed changes to molecular circuitry underlying stunting to gain increased precision in targeting specific pathways as new therapies. We focus on epigenetic profiling, specifically targeting histone modifications. Epigenetic profiling provides a powerful tool for studying stunting, as the epigenome is responsive to and reflects environmental signals [27]. This is because the enzymes that deposit and remove histone marks are dependent on the availability of key metabolites, the levels of which are reflected in epigenetic profiles [28], and furthermore, it has been suggested that the epigenome plays a role in intragenerational and transgenerational inheritance [29, 30]. While multiple studies have been conducted to understand the epigenetic changes associated with malnutrition, most of them have concentrated on DNA methylation [31–34] and histone modifications have been less well explored.

In our previous work, we showed that both H3 trimethylation on lysine 4 (H3K4me3, associated with active promoters [35]) and H3 acetylation on lysine 27 (H3K27ac, associated with active enhancers [36]) undergo large scale changes within the first year of life. We observed increased levels of activation at stress and immune response genes in 18-week-old children through hyperactivated H3K27ac. This was followed by massive changes in one-year-old stunted children in which H3K27ac was globally downregulated and H3K4me3 redistributed from promoters to distal ectopic sites. Both H3K4me3 and H3K27ac changes in one-year-olds point to downregulated immune responses and suggest alterations in one-carbon metabolism. In both cases we observed relatively modest changes in early infancy followed by large-scale changes at one year. H3K4me3 and H3K27ac are associated with active transcription and dynamic changes in response to environmental changes. This prompted us to explore the role of more stable chromatin structures in early infancy relative to the development of stunting. To address this, we performed chromatin immunoprecipitation followed by sequencing (ChIP-seq) in 18-week-old children targeting H3 trimethylation on lysine 9 (H3K9me3). H3K9me3 mark is associated with constitutive heterochromatin and has a role in development by repressing lineage inappropriate genes, as well as a role in repressing endogenous retroviruses (ERVs) and other transposable elements (TEs) [37, 38], whose regulatory roles in gene expression are currently being investigated [39–41]. Furthermore, animal studies suggest that H3K9me3 plays a crucial role in transgenerational inheritance in response to environmental changes, including nutritional changes [42, 43]. For this reason, we also profiled H3K9me3 in mothers to uncover relationships between maternal and child heterochromatin in stunting. The results suggest that globally elevated H3K9me3 in early infancy predisposes children to poor growth, possibly through suppression of TEs involved in development of immune responses to pathogens. The H3K9me3 pattern in early infancy is thus a predictor of the risk of stunting and supports a model in which the immune system of stunted children becomes compromised in early infancy.

## Results

### Study overview

**Fig. 1:**
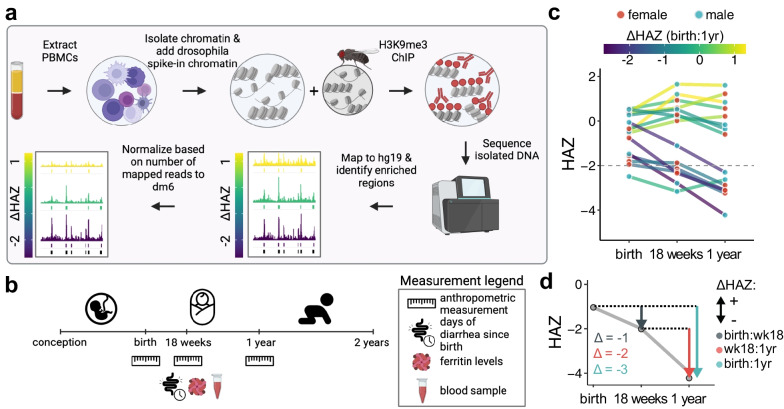
Project overview and experimental design. **a** Experimental overview. **b** The timeline of early infancy showing sample collection time point and available anthropometry. Blood samples were collected at 18 weeks of age. Additional information used in this study is highlighted in the diagram under the age of a child at the time of information collection. **c** Overview of the growth trajectories of children whose samples were used in this study. Grey points indicate HAZ score (y-axis) at a given age (x-axis), the growth trajectories are colored based on ΔHAZ between birth and one year, and *n* indicates the number of individuals. Children with HAZ scores at one year below the dashed line are classified as stunted. **d** ΔHAZ score explained. Grey line presents growth trajectory of a single individual and is created by connecting HAZ scores (y-axis) at given ages (x-axis). The colored arrows indicate changes in HAZ score between two time points, i.e., ΔHAZ. Dark grey—change between birth and 18 weeks, red—change between 18 weeks and one year, green—change between birth and one year

To identify genome-wide H3K9me3 changes associated with childhood stunting, we performed ChIP-seq targeting H3K9me3 in peripheral blood mononuclear cells (PBMCs) from 18-week-old infants (n = 15) from Bangladesh who were enrolled in the PROVIDE (performance of rotavirus and oral polio vaccines in developing countries) study (n = 700, ref. [44], Fig. 1a, Additional file 1: Table S1). While the blood samples come from 18-week-old infants, for each child we have anthropometric measurements including height-for-age z (HAZ) and weight-for-age z (WAZ) scores within the first year of the child’s life with additional biomarkers from 18 weeks (Fig. 1b, Additional file 1: Table S2). Each HAZ score provides a snapshot of a child’s current health status, which can change dynamically within the first year of life (Fig. 1c). In our analysis, we used the ΔHAZ score as a measure of growth change between two time points (Fig. 1d). Samples selected for this study reliably represent the PROVIDE spectrum of HAZ and ΔHAZ scores and are balanced in sex representation (Additional file 1: Fig. S1,2).

### H3K9me3 levels are correlated with poor growth

**Fig. 2:**
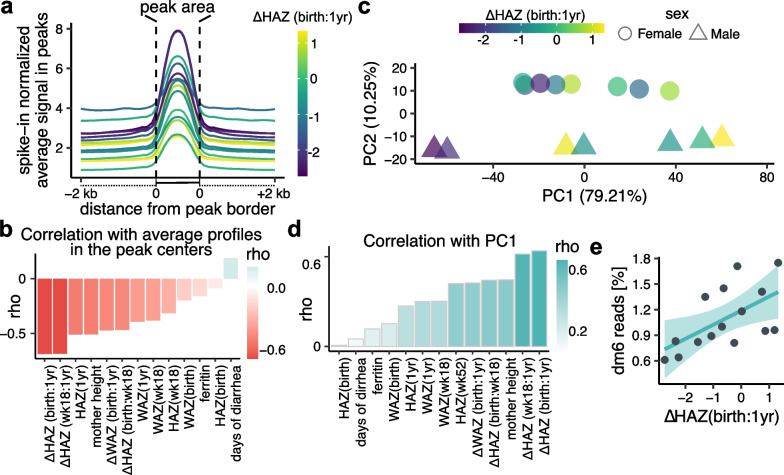
H3K9me3 profiles are globally correlated with negative growth change between birth and one year. **a** Average H3K9me3 profiles within peaks and 2 kb regions surrounding peaks. Each line represents an average profile of a child color coded by the child’s ΔHAZ (birth:1yr) score. The average profiles were normalized by drosophila spike-in normalization factors. **b** Spearman’s correlation coefficients (rho) between average normalized profile values from peak centers in (**a**) and a given measurement indicated on x axis. **c** PCA plot of H3K9me3 read counts in identified peaks. The numbers in brackets indicate variance explained by a given principal component. **d** Spearman’s correlation coefficients (rho) between PC1 and measurements indicated on x-axis. **e** Linear fit between ΔHAZ (birth:1yr) score and percentage of reads mapped to the *Drosophila melanogaster* genome. The relationship is significant (p-value = 0.021). Degrees of freedom = 13, adjusted R^2^ = 0.296

The average H3K9me3 signal within identified H3K9me3 enriched regions, i.e., peaks, was increased in children with poor growth between birth and one year as measured by the ΔHAZ (birth:1yr) score (Fig. 2a). And the pattern was conserved between the two sexes (Additional file 1: Fig. S3a,b). The ΔHAZ (birth:1yr) score showed the strongest correlation with the average H3K9me3 signal and H3K9me3 was negatively correlated with the ΔHAZ (birth:1yr) score (Fig. 2b). Furthermore, the first component (PC1) of principal component analysis (PCA) explained almost 80% of the variance in H3K9me3 peak signal (Fig. 2c) and again was strongly correlated with ΔHAZ (birth:1yr) score (Fig. 2d). While there were sex differences in the H3K9me3 profiles, these were associated with the second principal component (PC2) and explain only about 10% of the variance in the data. These results were not influenced by enzymatic cleavage (“clipping”) of histone H3 [45], or by global changes to histone H3 levels (Additional file 1: Fig. S4, Additional file 1: Supplemental text).

Both the average H3K9me3 profiles and signal within H3K9me3 peaks used for PCA were normalized based on *Drosophila* spike-in content, which we used in our experimental design to uncover any global H3K9me3 profile changes (Additional file 1: Fig. S5a). While normalizing the H3K9me3 profiles based on read count per million mapped reads still showed a negative correlation between a child’s ΔHAZ (birth:1yr) score and the overall H3K9me3 level, the pattern was much weaker and could have been easily missed without spike-in normalization (Additional file 1: Fig. S5b,c). Furthermore, we show that increased proportional content of *Drosophila* spike-in in the sequencing files, which suggests a genome-wide loss of H3K9me3 (Additional file 1: Fig. S5d), was significantly associated with ΔHAZ (birth:1yr) score (Fig. 2e, Additional file 1: Fig. S5e,f). Altogether, these results suggest that infants undergoing poor growth between birth and one year of age have globally elevated H3K9me3 levels compared to healthier infants.

### Differentially affected H3K9me3 regions are associated with stunting relevant pathways

**Fig. 3:**
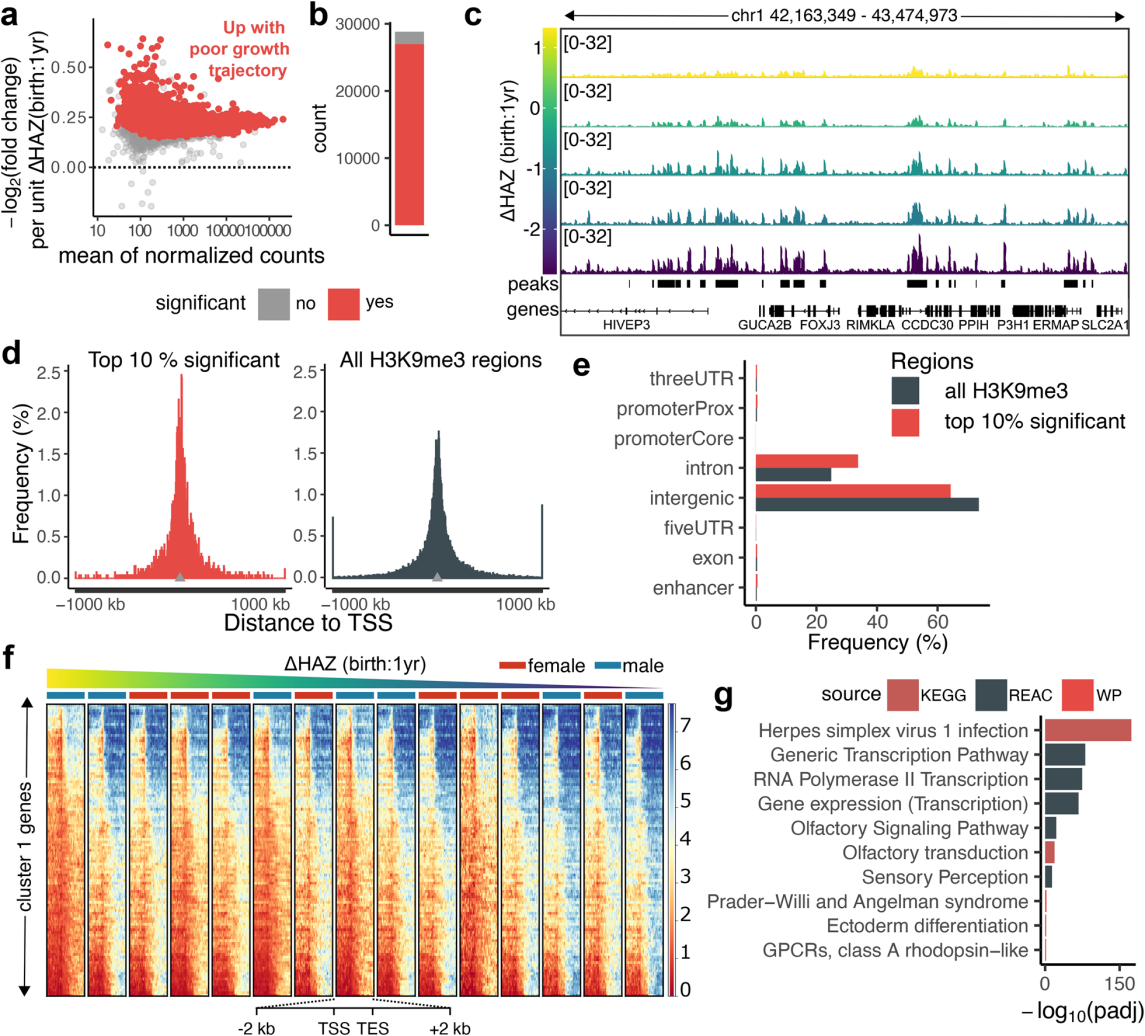
The most significant regions are nearby gene sites and highly enriched in viral infection pathways. **a** MA-plot shows changes of H3K9me3 levels with negative unit changes of ΔHAZ (birth:1yr) score. Each point is an H3K9me3 region, x-axis shows the mean normalized H3K9me3 coverage and y-axis shows −log_2_(fold change) of H3K9me3 coverage per unit increase of ΔHAZ (birth:1yr) score. Regions whose coverage increased in association with negative growth trajectory are above the x-axis. Regions with significant association (padj < 0.05) are highlighted in red. **b** Bar plot showing the number of H3K9me3 regions significantly associated vs. not significantly associated with ΔHAZ (birth:1yr) score in red and grey respectively. **c** Genome browser snapshot showing an example region, where normalized H3K9me3 coverage increased with poor growth trajectory indicated by low ΔHAZ (birth:1yr) score. **d** Histograms comparing the distances of the top 10% most significant (left) vs. all (right) H3K9me3 regions to TSSs. Shown are distances closer than ± 1 Mb from TSSs; more distal regions were accumulated into bins on both sides of the histogram. The distances from TSSs when not accounting for directionality are significantly higher within all H3K9me3 regions compared to the 10% significant regions (two sample t-test, H_0_: μ_top10_ = μ_all_, H_1_: μ_top10_ < μ_all_, *p* < 2.2e-16). **e** Bar plot showing relative distribution of the top 10% most significant vs. all H3K9me3 regions over genomic classes. The differences in the overlaps between the two classes are significant (Chi-square test, H_0_: the number of overlaps within the top 10% significant and all H3K9me3 regions are independent, *p* < 2.2e-16). **f** Heatmaps showing normalized H3K9me3 coverage over genes and ± 2 kb surrounding regions from cluster 1 identified in Additional file 1: Fig. S8. Heatmaps are organized based on child’s ΔHAZ (birth:1yr) score. **g** Results of gene enrichment analysis of cluster 1 genes shown in (**f**). Plotted are −log_10_ adjusted p-values (padj) of significant enrichments (padj < 0.05). Results come from three different sources: KEGG, Reactome (REACT), and WikiPathways (WP)

To gain insight into the roles of H3K9me3 regions in stunting, we performed differential analysis in which we asked how H3K9me3 levels changed at each peak with respect to ΔHAZ (birth:1yr) score while controlling for sex. The vast majority of the H3K9me3 regions were significantly upregulated in children with negative changes in HAZ scores during the first year (Fig. 3a–c, Additional file 2: Table S3), as expected given the significant global upregulation discussed above. The mean fold change per unit decrease of ΔHAZ (birth:1yr) score within significantly affected regions was 1.18 (Additional file 1: Fig. S6a), thus indicating an 18% change in the H3K9me3 signal per unit ΔHAZ (birth:1 yr) score. For functional analysis, we focused on the top 10% of the most significantly affected regions. These regions were not highly specific for a particular cell type (Additional file 1: Fig. S6b), and compared to the H3K9me3 regions overall, they are closer to genes (Fig. 3d), including being notably more enriched in intronic regions (Fig. 3e). The enrichment in intronic over intergenic regions is specific for the top 10% of the significant regions and the enrichment declines rapidly with further ranking (Additional file 1: Fig. S6c,d). We next asked if these regions are enriched with co-localizing histone marks and DNA-binding factors (DBFs) (Additional file 1: Fig. S6e). As expected, we found the highest enrichment was with the known sets of H3K9me3 regions, but also we found significant enrichment in enhancer regions marked by H3K27ac or H3K4me1 (histone H3 monomethylation on lysine 4) [46], and in H3K36me3 (histone H3 trimethylation on lysine 36). Colocalization of H3K9me3 with H3K36me3 has been shown to be crucial for repressing enhancer activity [47]. Interestingly, we further found significant enrichment in binding sites for the pioneer transcription factor *PU.1* (*SPI1*), a factor crucial for hematopoietic cell fate control [48, 49], suggesting that in stunting, increased H3K9me3 levels at these sites may prevent proper immune cell development through restricting *SPI1*/*PU.1* binding [50].

The assignment of these top H3K9me3 regions to their gene targets revealed genes that are involved in immune system processes, tissue development, nitrogen and phosphate metabolism and stress responses (Additional file 1: Fig. S7a). Importantly, mouse studies identified many of these gene targets to be involved in processes tightly associated with stunting (Additional file 1: Fig. S7b), such as altered interferon gamma (IFN-γ) levels [51], abnormal enterocyte proliferation associated with environmental enteric dysfunction (EED) [4, 17], or neurological pathways, such as altered post-tetanic potentiation, which potentially impacts neurocognitive development [5, 6].

### Genes associated with high H3K9me3 levels are highly enriched in viral immune response pathways

The proximity of these H3K9me3 regions to genes prompted us to explore the H3K9me3 patterns within genes. Two distinct clusters of genes were identified (Additional file 1: Fig. S8a). Cluster 1 contains a smaller subset of genes (n = 1258) whose coverage changed dynamically with ΔHAZ (birth:1yr) score (Fig. 3f), while the genes in cluster 2 had generally very low H3K9me3 coverage. The cluster 1 genes were enriched in transcription regulation, as well as nucleotide biosynthesis and metabolism, protein deubiquitination, and sensory development (Fig. 3g, Additional file 1: Fig. S8b). The most significantly enriched pathway, however, was Herpes simplex virus 1 infection. We explored the distribution of the genes of interest within this pathway and surprisingly found that 243 out of the 244 genes of interest are genes coding for zinc-finger antiviral proteins (*ZAP*s) that bind GC-dinucleotide enriched viral mRNA sequences and target them for degradation [52]. If increased H3K9me3 levels indicate repression of these genes, these results could suggest that one of the mechanisms through which H3K9me3 predisposes children to stunting is through paralyzing immune responses to viral invasion.

### Elevated H3K9me3 regions appear to mask TEs with potential regulatory roles in immune responses

**Fig. 4:**
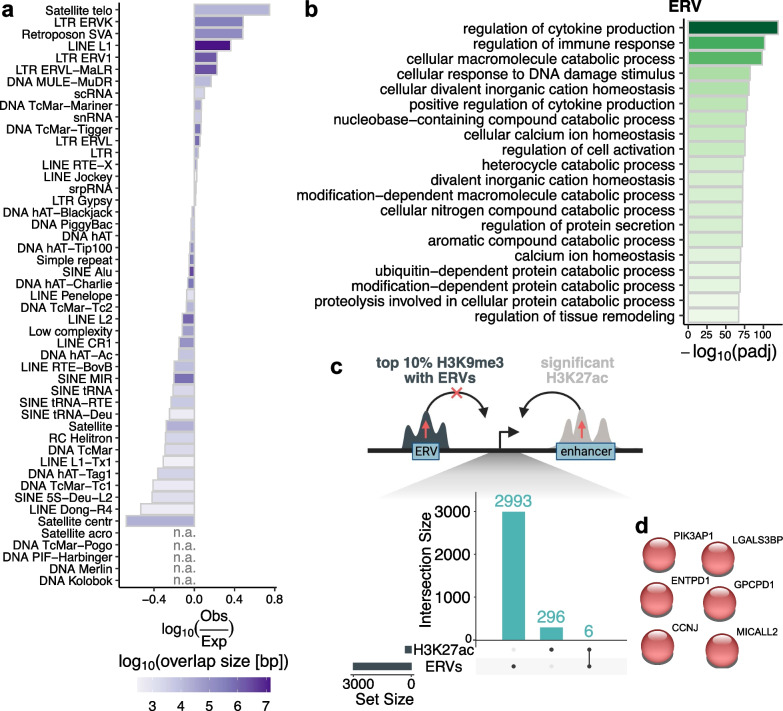
Misregulated H3K9me3 regions are enriched in transposable elements with potential roles in immune system modulation. **a** Ratio of observed over expected overlaps of TEs with top 10% significantly misregulated H3K9me3 regions with ΔHAZ (birth:1yr) score. Positive values indicate overlaps higher than expected. The color shows the total overlap size in base-pairs for a given TE class. **b** Top 20 GO:BP terms, as displayed by GREAT, associated with ERVs within the top 10% significantly affected H3K9me3 regions. Plotted are negative log_10_ FDR-corrected p-values (padj). Padj values < 0.05 were considered significant. **c** Comparison of gene targets shared between enhancers with increased H3K27ac values at 18-weeks in stunting [36] and ERVs within the top 10% significant regions with increased H3K9me3 levels as illustrated in the top part of the panel. **d** Shared target genes between H3K27ac and ERVs from **c**. No significant functional enrichment was identified

Since H3K9me3 is well known to localize to and suppress repeated sequences and various classes of transposable elements [37], we next explored the endogenous retroviruses (ERVs) and other classes of transposable elements (TEs) that overlapped with all significantly affected H3K9me3 regions (Additional file 1: Fig. S9a), as well as the top 10% of significant regions (Fig. 4a). In both sets, we identified similar overrepresented classes of TEs, with higher overrepresentation in the top 10% significant regions. We therefore focused on delineating the possible functional relevance of the highly overrepresented TE classes within the top 10% significant regions, specifically the ERVs, long interspersed nuclear elements-L1 (LINE L1), SINE-R-VNTR-*Alu* (SVA) retrotransposons, and telomeric satellite DNA. Overall, we observed highly significant overlap of ERVs (Fig. 4b) and LINE L1 (Additional file 1: Fig. S9b) with regulatory regions for genes implicated in immune response pathways, including cytokine production and immune cell activation, as well as in DNA damage response pathways, catabolic pathways, and cation homeostasis pathways. Interestingly, we also noted an enrichment of H3K9me3 regions overlapping SVA retrotransposons in nutrient sensing pathways (Additional file 1: Fig. S9c), however, with much lower significance compared to the enrichment of pathways relevant to ERVs and LINE L1. Telomeric satellite DNA associated with H3K9me3 was also modestly associated with genes implicated in adhesion (Additional file 1: Fig. S9d).

The results from these analyses suggest that high H3K9me3 levels at these gene regulatory sites suppressed immune responses. We previously reported increased H3K27ac targeting genes associated with viral infection [36]. We therefore tested whether ERVs within the top significant H3K9me3 regions share common gene targets with significantly upregulated H3K27ac regions (Fig. 4c). The majority of gene targets were specific to ERVs or H3K27ac, except for 6 genes that, however, did not show any significant pathway enrichment (Fig. 4d). Expanding the analysis to ERV regions overlapping all significant H3K9me3 regions increased the number or overlapping genes to 61, but even these were not associated with any specific pathways.

### Analysis of maternal H3K9me3 profiles

**Fig. 5:**
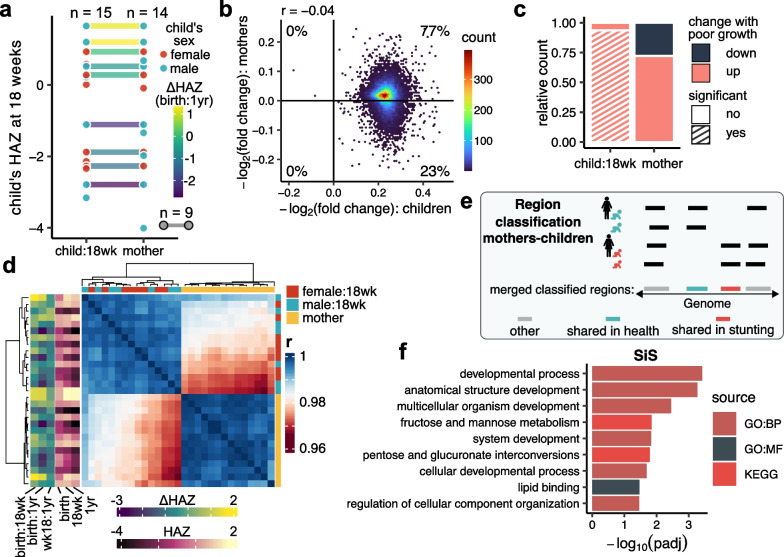
Relationships between H3K9me3 profiles of mothers and 18-week-old infants. **a** Overview of samples used in this study. Each dot represents an individual whose sample was used in this study. X-axis separates 18-week-old children from mothers, y-axis represents child’s HAZ score at 18 weeks of age. A link is drawn only between matching mother–child pairs and is colored based on child’s ΔHAZ (birth:1yr) score. The three different *n*s indicate the number of 18-week-old children (left), mothers (right) and matching mother–child pairs (bottom legend). **b** Comparison of differential analysis results showing −log_2_(fold changes) per unit of child’s ΔHAZ (birth:1yr) score. On the x-axis are plotted results for 18-week-old children, on the y-axis results for mothers. Each point is an H3K9me3 region identified both in mothers and 18-week-old children. Positive values indicate increasing H3K9me3 levels in association with poor growth trajectory. The color coding shows the density of the points. The numbers indicate the percentage of points falling into a given quadrant. **c** Bar plot summarizing results of differential analysis from 18-week-old children vs. mothers. Regions classified as going up with poor growth trajectory are those with positive −log_2_(fold changes) per unit of child’s ΔHAZ (birth:1yr) score and vice versa. **d** Heatmap showing Pearson’s correlation coefficients between H3K9me3 profiles of all 18-week-old children and all mothers whose samples were used in this study. **e** Illustration showing classification of regions. Regions identified only in mothers of healthy children (green icon) and in healthy children are classified as SiH (green). Regions identified only in mothers of stunted children (red icon) and in stunted children are classified as SiS (red). **f** Gene set enrichment results for genes associated with SiS regions. Plotted are −log_10_(padj) values of significant enrichments (padj < 0.05). Results come from three different sources: KEGG, GO:MF, GO:BP

To determine the relationship between maternal and child’s H3K9me3 profiles, we performed H3K9me3 ChIP-seq on PBMC samples from mothers (n = 14, Fig. 5a) enrolled in the same PROVIDE study. Through differential analysis, we first explored which H3K9me3 regions were misregulated in relationship to the child’s ΔHAZ (birth:1yr) score while controlling for the child’s sex (Additional file 1: Fig. S10a, Additional file 3: Table S4). While we did not observe any significant relationship between maternal H3K9me3 and a child’s ΔHAZ (birth:1yr) score, or a strong correlation between the differential analysis results from children and mothers (Fig. 5b), we noticed that the majority of the maternal H3K9me3 regions were upregulated in mothers whose children had low ΔHAZ (birth:1yr) scores, partially capturing the global pattern observed in 18-week-old infants (Fig. 5c, Additional file 1: Fig. S10b). Further exploration of global relationships between H3K9me3 profiles of mothers and children through correlation analysis of H3K9me3 profiles resulted in identification of two distinct clusters separating mothers from infants (Fig. 5d) suggesting that H3K9me3 carries strong age-related signatures that are possibly masking weaker signatures of stunting. Interestingly, PCA separated maternal and infant samples with PC2, while PC1 still captured 81% of the variance in the data and preserved the strong relationship of the infant H3K9me3 profiles to ΔHAZ (birth:1yr) score (Additional file 1: Fig. S10c,d). The separation of maternal H3K9me3 profiles along PC1 did not reveal a strong correlation with any of the biomarkers (Additional file 1: Fig. S10d). Furthermore, narrowing focus to only the top 10% of the misregulated regions in stunted children did not reveal any new patterns in comparison to the analysis performed using all H3K9me3 regions (Additional file 1: Fig. S10e,f).

To determine if subtler patterns in the maternal H3K9me3 profiles are potentially contributing to stunting, we tested whether there were regions shared only between mothers of stunted children and stunted children, here termed shared in stunting (SiS). We also tested whether likewise there were maternal H3K9me3 regions in mothers of healthy children that were identified in healthy children (SiH) (Fig. 5e). We identified 110 SiS and 97 SiH H3K9me3 regions (Additional file 1: Fig. S11a) that were not cell-type specific (Additional file 1: Fig. S11b) and did not have a distinct signature in terms of distribution across the genome (Additional file 1: Fig. S11c,d). However, functional annotation revealed a distinct set of pathways specific for each region set. SiS regions were associated with genes involved in multiple developmental processes, lipid binding, and sugar metabolism, specifically fructose and mannose metabolism, and pentose and glucuronate interconversions (Fig. 5f). In contrast, SiH regions were associated with pathways involved in signaling, regulation, development, growth factor regulation, and endothelial cell migration (Additional file 1: Fig. S11e).

### Histone marks misregulated in one-year-old stunted children often reside within H3K9me3 regions

**Fig. 6:**
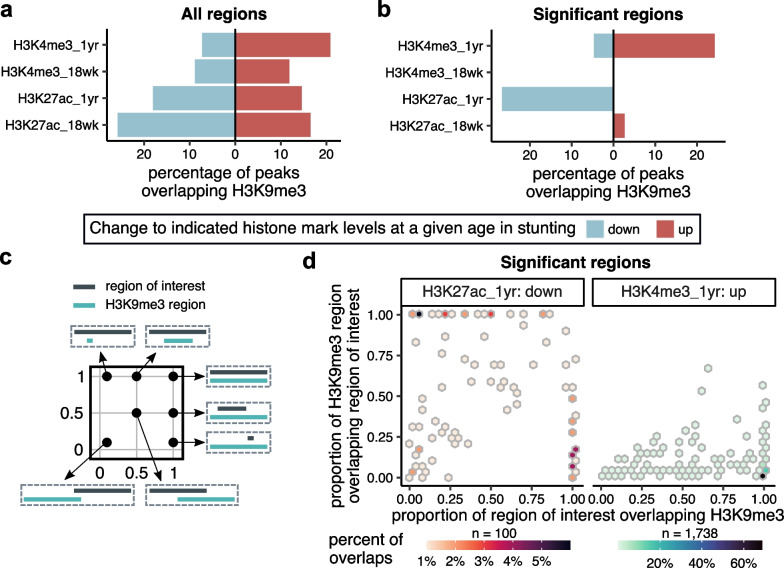
H3K9me3 regions upregulated in 18-week-old infants overlap active regulatory elements misregulated in 1-year-old stunted children. **a** Bar plot showing percentage of all H3K4me3 and H3K27ac regions identified in 18-week- and one-year-old children that overlap H3K9me3 regions identified in 18-week-old infants. The overlaps were counted separately for upregulated (red) vs. downregulated (blue) regions at a given age in stunting. **b** Bar plot as in (**a**) only showing overlaps with regions that were significantly different in stunted children. **c** Explanatory illustration for panel (**d**) showing different overlap scenarios. **d** The plots show the proportions of H3K9me3 regions that overlap significantly downregulated H3K27ac (left) or significantly upregulated H3K4me3 (right) in one-year-old children (x-axis) versus the proportions of these H3K27ac and H3K4me3 regions that overlap with H3K9me3 (y-axis). Color coding indicates the percentage of regions falling into a given bin. The total number of overlapping regions for each category is indicated above the individual color bars

To gain more comprehensive insight into epigenetic changes associated with stunting, we combined H3K9me3 results from this study with previously published results describing H3K4me3 [35] and H3K27ac [36] changes associated with stunting in 18-week-old and one-year-old children from the same cohort (but not all the same individuals; Additional file 1: Fig. S12a-d). First, we identified overlaps between H3K9me3 regions and H3K4me3 or H3K27ac regions in both age groups (Fig. 6a). While we observed a decrease in the overlap of H3K9me3 and H3K27ac peaks between 18 weeks and one year, there was an intriguing increase in the overlaps between H3K9me3 and H3K4me3 regions during infancy, especially in the H3K4me3 regions that were upregulated in stunted children. By further narrowing our focus on significantly affected H3K4me3 and H3K27ac regions in stunting, we found that in 18-week-old children, these regions are almost exclusively distinct from the H3K9me3 regions, compared to the significantly affected H3K4me3 and H3K27ac regions in one-year-old children that tended to colocalize with H3K9me3 signal in 18-week-old children (Fig. 6b, Additional file 1: Fig. S12e-h). Notably, there was an overlap of almost 25% of the small upregulated H3K4me3 regions that were previously suggested to be redistributed from the larger H3K4me3 regions at TSSs [35], and nearly 30% of the H3K27ac regions downregulated in one-year-old stunted children overlapped with H3K9me3 peaks. The downregulated H3K27ac regions in one-year-old stunted children did not have a very specific location relatively to the overlapping H3K9me3 regions, while the upregulated H3K4me3 regions in one-year-all children were predominantly encompassed by H3K9me3 regions (Fig. 6c,d, see Additional file 1: Fig. S13a,b for overlaps with all H3K4me3 and H3K27ac regions). These observations suggest a possible mechanism in which the methylation machinery compensates for the high levels of H3K9me3 observed in 18-week-old infants through increased H3K4me3 levels at one year of age.

Functional annotation of overlaps with H3K4me3 regions upregulated in stunted children at one year suggests that these are involved in regulating transcription, cell adhesion and developmental processes (Additional file 1: Fig. S14a), and there was also a relatively weak colocalization of these regions with H3K36me3 (Additional file 1: Fig. S14b). In contrast, the overlaps with H3K27ac peaks that were downregulated in stunting at one year showed enrichment only in cell activation pathways (Additional file 1: Fig. S14c), in addition to strong colocalization with both activating and repressing histone marks (Additional file 1: Fig. S14d), as well as with a broad range of DBFs (Additional file 1: Fig. S14e).

## Discussion

This study was motivated by two goals. The first goal was to uncover epigenetic changes in early infancy associated with the development of stunting. This goal arose from our previously published results in which we observed large-scale stunting-associated changes to H3K4me3 [35] and H3K27ac [36] in one-year-old children suggesting immune system exhaustion and alterations to one carbon-metabolism. However, in those studies we saw no significant changes to H3K4me3 in 18-week-old infants and only relatively smaller changes to H3K27ac which suggested activation of stress responses and genes involved in viral infection. We hypothesized that if there were epigenetic changes in early infancy predisposing children to become stunted, we would need to investigate a more stable mark associated with development, such as H3K9me3 [53]. The second goal was to determine if there were relationships between maternal and child H3K9me3 profiles that could shed light on the propagation of stunting, including transgenerational inheritance, which contributes to the development of stunting via H3K9me3 and other factors in animal models [42].

**Fig. 7:**
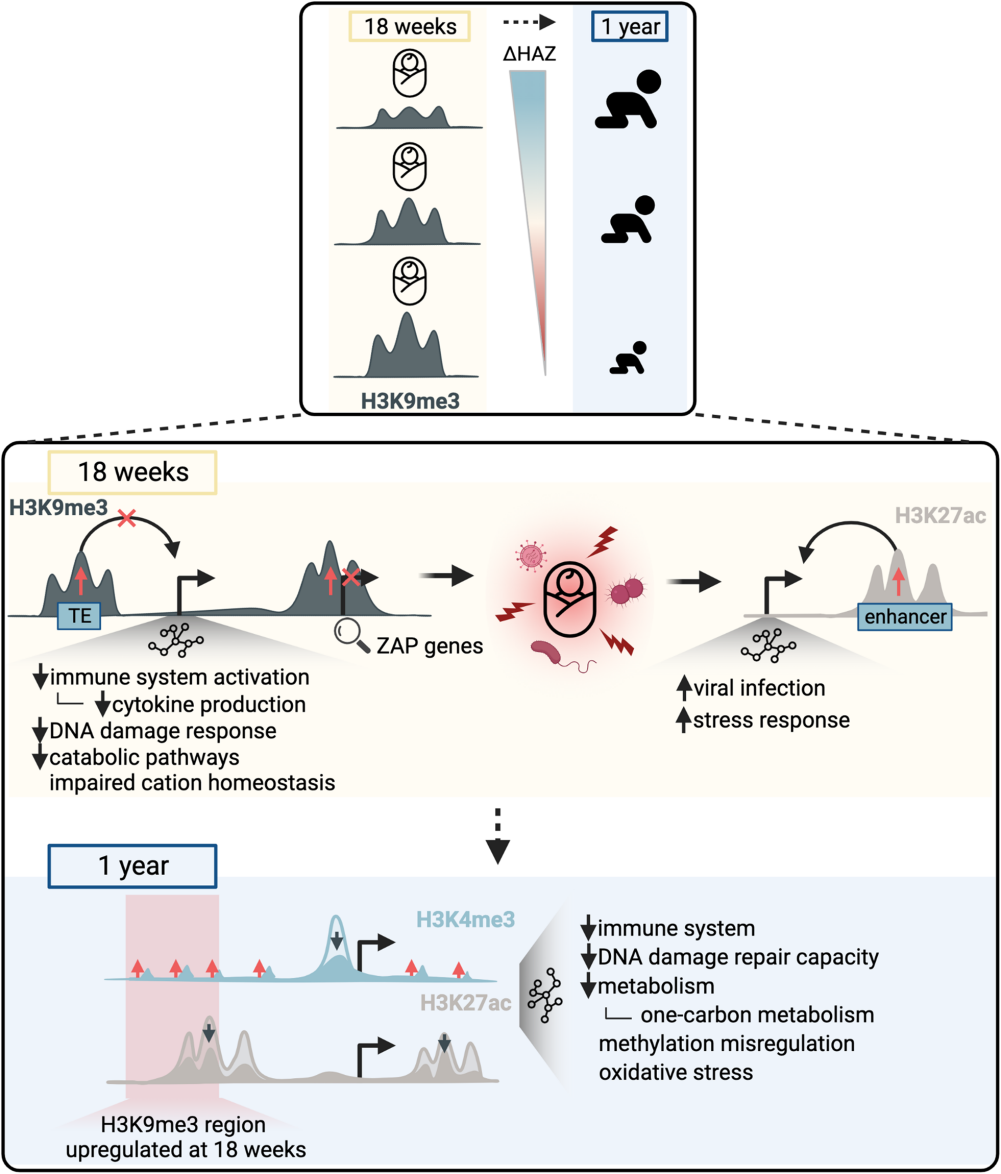
Summary and working model. Top panel shows that globally increased H3K9me3 levels at 18 weeks of age are associated with poor growth through the model illustrated in the bottom panel. In this model, increased H3K9me3 levels restrict expression of *ZAP* genes and regulatory activity of key TEs resulting in immature immune responses, and thus high pathogen load and overall stress in children destined to be stunted. These changes then trigger increased stress responses and activation of pathways involved in viral infection as indicated by elevated H3K27ac. As an adaptation to long-term stresses, H3K4me3 and H3K27ac undergo large-scale changes, as described previously [35, 36], which affect the highlighted pathways by one year of age. A significant proportion of H3K4me3 and H3K27ac changes observed in one-year-old children fall into misregulated H3K9me3 regions identified in 18-week-old children

The results presented here along with prior work on H3K4me3 and H3K27ac are summarized in Fig. 7. The major finding here is that globally elevated H3K9me3 levels in 18-week-old infants are associated with poor growth between birth and one year of age as indicated by the ΔHAZ (birth:1yr) score (Fig. 2). Notably, H3K9me3 levels show much weaker association with a child’s current health status in terms of the HAZ score at 18 weeks (Fig. 2), suggesting that altered H3K9me3 levels do not reflect immediate changes in a child’s environment, but instead may play a role in programming developmental pathways over time. Overall, these results suggest the possibility that high H3K9me3 levels could be used as a biomarker for poor growth before the overt manifestation of stunting. We attempted to confirm the changes in H3K9me3 by Western blotting but found that there is a high degree of variability in the values measured by this method relative to the change in H3K9me3 detected by spike-in normalization (Additional file 1: Fig. S4b-d).

Through the detailed analyses presented here, we explored molecular mechanisms through which elevated H3K9me3 levels may predispose children to poor growth. Genes residing within misregulated H3K9me3 regions were involved in Herpes simplex virus 1 infection pathway (Fig. 3f,g). These include a large number of *ZAP* genes which encode proteins that target viral mRNA for degradation [51]. Thus, their possible repression resulting from elevated H3K9me3 potentially predisposes children to be more vulnerable to viral infection. TEs within the top significant H3K9me3 regions may also contribute to H3K9me3 regulatory mechanisms. While the functional role of TEs is largely unexplored, it has been thought that TEs, such as ERVs, are marked by H3K9me3 to ensure they remain transcriptionally silent. Recent studies also show that TEs can act as binding sites for transcription factors (TFs) [54] and furthermore that they have roles in development of innate immune responses [39]. Here we show that TEs residing within the top significant H3K9me3 regions are in proximity to genes involved in immune system activation, where we observed an especially high enrichment in cytokine production pathways, as well as DNA damage pathways, catabolic pathways, and other pathways with less significant enrichment (Fig. 4b, Fig S9b-d). It has been recently shown that non-repressed ERVs can create competition for TF binding, and in addition, decreased heterochromatin levels at ERVs were associated with overall decreased H3K27ac in other genomic regions [41]. This model fits well with our data in which increased H3K9me3 at ERV sites and increased H3K27ac levels at other sites in the genome suggest deleterious masking of ERV sites. Taken together, these results suggest a model (Fig. 7) in which increased H3K9me3 levels at key TEs and *ZAP* genes may paralyze activation of immune system responses in early infancy, leading to an increased pathogen load and overall stress in children destined to become stunted. This is consistent with the apparent activation of genes associated with viral infection and stress response pathways through elevated H3K27ac.

Our results show that high levels of H3K9me3 in 18-week-old infants are associated with poor ΔHAZ (birth:1 yr) growth score and potentially hamper cytokine production. A recent study performed using samples from the same cohort shows that by one year of age stunting is inversely associated with high cytokine production, specifically of interleukin 8 (IL8) and transforming growth factor-β (TGFβ) [55]. The combined results are consistent with the emerging understanding that changes to immune system function are highly dynamic during early development [55], and further suggest that the development of a healthy immune system may involve precise timing in the developmental capacities of specific immune responses in response to pathogen burden.

We also discovered that the top differentially affected H3K9me3 regions are enriched in *SPI1*/*PU.1* TF binding sites (Additional file 1: Fig. S6e). *SPI1*/*PU.1* is a TF crucial for immune cell differentiation [48, 49] and for this reason we posit that potential H3K9me3-mediated repression of these sites leads to immune cell immaturity in early infancy. The gene targets of the top differentially affected H3K9me3 regions were further identified in pathways associated with altered circulating IFN-γ levels. Interestingly, these have been previously associated with stunting and susceptibility to amebiasis [51].

Integrated analysis with H3K4me3 [35] and H3K27ac [36] results from children from the same cohort (but not all the same individuals) further showed that, as expected, there was not much of overlap between the H3K9me3 regions and the activating H3K4me3 and H3K27ac marks at 18 weeks of age. However, a relatively high proportion of significantly misregulated H3K4me3 and H3K27ac regions in one-year-old stunted children overlap with the upregulated H3K9me3 regions at 18-weeks (Fig. 6b). About 25% of distal ectopic H3K4me3 regions occur within H3K9me3 regions and almost 30% of the upregulated H3K27ac regions overlap with H3K9me3 regions. The misregulated regions were previously described by gene association analysis to regulate processes highlighted in Fig. 7. These findings suggest a possible model in which elevated H3K9me3 levels in 18-week-old children preset a detrimental chromatin environment and that predisposes stunted children to mislocalization of H3K4me3 and misregulation of H3K27ac by one year of age. The H3K4me3 pattern is perhaps indicative of a compensating mechanism for previous over-suppression of these important regulatory regions.

We did not identify any significant H3K9me3 regions in mothers with a strong relationship to the child’s ΔHAZ (birth:1yr) score. However, the overall directionality of the H3K9me3 changes was in concordance with changes observed in the 18-week-old infants (Fig. 5b,c, Additional file 1: Fig. S10b). This suggests that there may be a relationship between the maternal and child profiles, but one that would probably require a much larger sample size to detect. Additional insight would likely come from analysis of paternal data, however, at the moment we are limited in both the number and types of samples available from this highly vulnerable population.

Overall, this study illustrates the power of epigenomic analyses to provide insight into the etiology of stunting, even when the sample numbers are limited and there are many contributing factors to its development.

## Conclusions

Analysis of the heterochromatin-associated histone mark H3K9me3 in PBMCs of 18-week-old infants showed that globally elevated H3K9me3 levels are associated with poor growth between birth and one year. Importantly, these changes were detectable before the overt appearance of the stunted phenotype and therefore present an opportunity for future work aimed at understanding mechanisms that may be manipulated to avert stunting as well as development of new biomarkers to detect it early. Moreover, these results suggest a relationship between H3K9me3 and the immune system dysfunction that develops in stunted children.

## Methods

### H3K9me3 ChIP-seq of human PBMCs

Deidentified PBMC samples from 18-week-old children (n = 15) and mothers (n = 14) enrolled in the PROVIDE study (n = 700) [44] were obtained in collaboration with icddr,b (International Centre for Diarrhoeal Disease Research, Bangladesh) in Dhaka, Bangladesh. The number of samples was established based on previous work [35, 36], and samples were selected to represent the PROVIDE spectrum of HAZ and ΔHAZ scores, as well as to be balanced in sex representation (Additional file 1: Fig. S1,2). ChIP-seq was performed as previously described in detail in [35]. Briefly, PBMC samples were fixed with formaldehyde, chromatin isolated and sheared, 0.02% (microgram/microgram) *Drosophila melanogaster* chromatin (cat #53083, Active Motif, Carlsbad, CA, USA) was added for spike-in normalization [56]. H3K9me3 antibodies (12.5 μl per 100 μg chromatin protein solution, Abcam Cat# AB8898, RRID: AB_30684) were used for DNA fragment selection by immunoprecipitation. Sequencing libraries were constructed using the Illumina TruSeq ChIP Library Preparation Kit following the manufacturer instructions. Libraries were sequenced using an Illumina NextSeq500 instrument with high-capacity cartridge in the University of Virginia Genome Analysis and Technology Core, RRID:SCR_018883, yielding between 52 to 142 million 150 bp single-end reads per sample (Additional file 1: Table S1).

### Western blotting

Western blotting was performed as previously described in [35]. 5ug of protein from 18 weeks of age was separated on a 4–20% gradient gel and processed for immunoblotting, using anti-H3K9me3 (Abcam Cat# AB8898, RRID: AB_30684) and then stripped and reprobed for total H3 c-terminus (Active Motif Cat# 39451, RRID: AB_2793242). Secondary: ECL Plex anti-rabbit IgG-Cy5 (Cytiva Cat# PA45011, RRID: AB_772205). Blots were imaged on Typhoon Cy5 600 PMT, Red (633) laser, 670 BP 30 filter.

### H3K9me3 ChIP-seq data preprocessing

Bowtie2 (2.2.6) [57] with default settings was used to map raw FASTQ files to the hg19 reference genome obtained with refgenie (0.9.3) [58]. SAMtools (0.1.19-44428cd) [59] were used to convert alignment SAM files to BAM format (function: *view -S -b*), sort (function: *sort*), and index (function: *index*) BAM files, report number of mapped (function: *view -c -F 4*) and unmapped (function: *view -c -f 4*) reads and to filter out unmapped reads (function: *view -h -F 4 -b*). ENCODE blacklisted sites [60] were removed with bedtools (v2.26.0) [61] *intersect* function with argument *-v*. The quality of both raw FASTQ files and final BAM files was checked with FastQC (v0.11.5) [62] together with multiQC (1.11) [63]. To further visually inspect the quality of the data, BAM alignment files were converted to bigWig files (initially without normalizing) and browsed with Integrative Genomics Viewer (IGV_2.7.2) [64]. To generate bigWig files, BAM files were first converted to BED files with coverage information using bedtools *bamtobed*, these files were then converted to COV files using bedtools *genomecov*, and finally bigWig files were generated from the COV files with UCSC *bedGraphToBigWig* (v4) [65]. Chromosome sizes for bedtools *genomecov* and *bedGraphToBigWig* were obtained with refgenie (0.9.3) [58].

Regions of enrichment, i.e., peaks, were generated from BED coverage files using SICER (v1.1) [66] against input (separate input files for children and mothers) with *redundancy threshold* = 1, *window size* = 1000, *fragment size* = 425, *effective genome fraction* = 0.74, *gap size* = 5000, and *FDR* = 0.01.

Count tables with the number of mapped reads for each identified region were created for children and mothers separately. Identified peaks were first merged (mothers and children separately) with bedtools *merge* and quantification was done with bedtools *multicov* using BAM alignment files and merged regions.

Code is available from: https://github.com/AubleLab/H3K9me3_stunting.

### Normalization

Drosophila spike-in reads were used for normalization to account for possible global changes in H3K9me3 levels (Additional file 1: Fig, S5a). To retrieve the number of spike-in reads within each sample, raw FASTQ files were mapped to the *Drosophila* dm6 reference genome (obtained with refgenie) using bowtie2 with default parameters. SAMtools (0.1.19-44428cd) [59] were used to convert alignment reads to BAM format (function: *view -S -b*), sort (function: *sort*), and index (function: *index*) BAM files, ENCODE blacklisted sites [60] were removed with bedtools (v2.26.0) [61] *intersect* function with argument *-v*, and finally SAMtools were used to report number of mapped reads (function: *view -c -F 4*). The final normalization factors were then determined by dividing the number of reads that mapped to dm6 by an arbitrary constant (500,000). These normalization factors were then used in differential peak analysis with DESeq2 (1.30.1) [67]. Inverted values of the normalization factors (1/NF) were used to generate normalized bigWig files, specifically bedtools *genomecov* function with argument *-scale* set to the inverted normalization factor was used to create normalized bedGraph files from BAM alignment files, which were further converted to normalized bigWig files with UCSC *wigToBigWig* tool (v4) [65].

Correlations between the percentage of reads mapped to the dm6 reference genome (indicative of globally misregulated H3K9me3 levels, see Additional file 1: Fig. S5d) with all available biomarkers/measurements were calculated using R *cor* function with *method* = *“spearman”*.

The relationship between the percentage of reads mapped to dm6 and a child’s ΔHAZ (birth:1 yr) score was determined by fitting a linear model with the R *lm* function, where the p-value < 0.05 was considered significant.

### Differential analysis

DESeq2 (1.30.1) [67] was used to identify differentially regulated H3K9me3 regions in both child and maternal datasets. Raw count tables along with drosophila normalization factors were passed to DESeq2 with a formula set to child’s sex + child’s ΔHAZ (birth:1 yr) score. Regions with FDR corrected p-values (padj) less than 0.05 were identified as significantly different. Other combinations of formulas combining a child’s sex with other available biomarkers were tested (data not shown), however, no significant regions were identified.

### PCA

PCA was performed using *prcomp* R function applied to spike-in- and regularized logarithm- (DESeq2 (1.30.1) [67] *rlog* function) normalized count tables. Counts from all available regions were used for PCA. Results were plotted using ggplot2 (3.3.6) [68] and points colored based on child’s ΔHAZ (birth:1 yr) score with a palette from viridis package (0.6.2) [69]. PCA performed on both children’s and maternal data together was applied on spike-in-normalized counts over merged (bedtools *merge* function) children’s and maternal H3K9me3 regions. Correlations between PC1 with all available biomarkers/measurements were calculated using R *cor* function with *method* = *“spearman”*.

### Average profiles

Average spike-in-normalized H3K9me3 profiles were obtained by first using the deepTools (3.3.1) [70] *computeMatrix* function to quantify spike-in-normalized bigWig files over H3K9me3 regions identified in children’s datasets. The *computeMatrix* parameters were set to *scale-regions* mode with -*a 2000*, and -*b 2000* parameters to expand average profiles by additional ± 2 kb surrounding the H3K9me3 regions. From *computeMatrix* output, the values for average profiles were obtained with the deepTools *plotProfile* function with the *outFileNameData* parameter included, to get the actual average profile values in addition to the figure output. The resulting average profile values were then plotted using ggplot2 (3.3.6) [68], where smoothing of the profiles was done with *geom_smooth* function with parameters set to *method* = *"loess", span* = *0.2* and colored based on child’s ΔHAZ (birth:1 yr) score using viridis package (0.6.2) [69].

Average profiles using read count per million mapped reads normalization were obtained by mapping BAM alignment files onto the H3K9me3 regions with ngs.plot (2.61) [71] with default settings, which automatically returns average profiles normalized to read count per million mapped reads. These profiles were then replotted with ggplot2 to include coloring based on a child’s ΔHAZ (birth:1 yr) score with viridis package.

Correlations between the centers of average profiles with all available biomarkers/measurements were calculated using the R *cor* function with *method* = *“spearman”*.

### Functional annotation of children’s H3K9me3 regions

The top 10% of H3K9me3 regions (based on FDR-corrected p-value, followed by p-value) that were identified in children’s datasets as differentially misregulated versus child’s ΔHAZ (birth:1 yr) score were compared with all of the H3K9me3 regions identified in the children’s datasets using GenomicDistributions (1.5.5) [72]. C*alcFeatureDistRefTSS* function with default parameters was used to compare the distances of the regions to the nearest TSSs (transcription start sites), and the distances were plotted with *plotFeatureDist* function with parameters *featureName* = *"TSS", size* = *1000000, infBins* = *T, nbins* = *300*. *CalcPartitions* function was used to identify overlaps with genomic classes. The genomic classes used as an input to the function were the default classes provided by the GenomicDistributions package with PBMC enhancer regions added from EnhancerAtlas 2 [73]. To create a list of PBMC enhancer regions, enhancer regions from relevant cell types were downloaded from EnhancerAtlas 2, namely CD4+, CD8+, CD14+, CD19+, CD20+, GM10847, GM12878, GM12891, GM12892, GM18505, GM18526, GM18951, GM19099, GM19193, GM19238, GM19239, GM19240, and PBMC cells. Finally, the regions were merged using the bedtools (v2.26.0) [61] *merge* function. The parameters to the *calcPartitions* function were set to *bpProportion* = *T*, and the overlaps were plotted with *plotPartitions* function with default parameters. The cell-type specificity of the regions was determined by using *calcSummarySignal* function with provided H3K9me3 cell-type specific signal matrix (see “Normalized cell-type specific H3K9me3 signal matrix” section of Methods) and plotted with *plotSummarySignal* function with parameters *plotType* = *"violinPlot", metadata* = *cellTypeMetadata, colorColumn* = *"tissueOfOrigin"*. A modified version of *calcCumulativePartitions* function was used to calculate cumulative distribution of the overlaps with genomic classes. The original function sorts regions based on their size; the modified function accepts presorted regions. The genomic classes used were identical with the ones used in *calcPartitions* function. The log_*2*_(fold change) of cumulative distribution values of intergenic/intronic regions were calculated and smoothed for plotting using R *loess* function with parameter *span* = *0.15*.

Histone mark and DBF (DNA-binding factor) enrichment of the top 10% significantly misregulated regions in children was calculated using LOLA (1.20.0) [74] against the CISTROME [75, 76] database of histone marks and transcription factors (here referred to as DBFs) filtered for blood cell types. The *userUniverse* parameter in LOLA was set to all defined children’s H3K9me3 regions, otherwise all default parameters were used. Significant enrichments were defined as those with q-values < 0.05. The most significant cell-type/antibody combinations were plotted in form of a dot plot, where dot sizes and dot colors reflect the significance of the enrichment for a given cell-type/antibody combination. Note that CISTROME provides only regions for the hg38 genome assembly, therefore all H3K9me3 regions used here were first converted to hg38 using UCSC *liftOver* tool [65].

Gene set enrichment of the top 10% significantly misregulated regions in children was done with GREAT [77]. Results were then reported as bar plots. Due to a larger number of identified GO:BP (Gene Ontology: Biological Process) terms, those were not reported in the bar plot and instead were first processed with REVIGO [78] with default parameters. The resulting network was further reorganized in Cytoscape (3.8.0) [79] and terms were manually classified into groups.

### Normalized cell-type specific H3K9me3 signal matrix

A matrix containing normalized H3K9me3 values across different cell types was used to infer cell-type specificity of regions of interest with GenomicDistributions (1.5.5) [72] *calcSummarySignal* function. To create the H3K9me3 cell-specific matrix, ENCODE [80] data was used. First, BAM alignment files from all available H3K9me3 ChIP-seq experiments on primary cells were used (ENCODE filters: Assay title—Histone ChIP-seq/Target of assay—H3K9me3/Organism—*Homo sapiens*/Biosample classification—primary cell/Genome assembly—hg19/Available file types—BAM). The downloaded BAM files were first sorted and indexed with SAMtools (0.1.19-44428cd) [59] *sort* and index *functions*, respectively. To define H3K9me3 regions across genome, BED files were also obtained from ENCODE (ENCODE filters: Assay title—Histone ChIP-seq/Target of assay—H3K9me3/Organism—*Homo sapiens*/Biosample classification—primary cell/Genome assembly—hg19/Available file types—BED narrowPeak). BED files were sorted (*sort -k1,1 -k2,2n*) and merged with bedtools (v2.26.0) [61] *merge* function. Raw signal values were quantified by using bedtools *multicov* function on H3K9me3 processed BAM files with merged H3K9me3 BED file. The resulting raw count tables were further processed in R. First, samples from the same cell-type origin were merged by summing the cell-type replicate signal values, followed by normalization of the signal values for individual cell types. The normalization was performed in three steps: i) signal values above the 99^th^ percentile for a given cell type were set to 1; ii) signal values below the 99^th^ percentile for a given cell type were normalized to range between 0 and 1 using the following formula: ${n}_{i}=\frac{{x}_{i}-min\left(x\right)}{max\left(x\right)-min\left(x\right)}$, where *n*_*i*_ is the normalized signal value for region *i*, *x*_*i*_ is the raw signal value for a region *i*, and *x* is a vector of raw signal values for a given cell type; and iii) signal values ranging between 0 and 1 within a given cell type were further quantile-normalized with *normalize.quantiles* function from preprocessCore package (1.52.1) [81].

#### Gene coverage heatmap

Heatmaps with spike-in-normalized coverage over genes were generated using deepTools (3.3.1) [70] *computeMatrix* function with *scale-regions* mode, and parameters –*sortRegions descend*, *-b 2000, -a 2000*. Mapped were spike-in-normalized bigWig files onto a BED file containing hg19 coordinates of protein-coding genes along with information about strandness. The gene coordinates were obtained with EnsDb.Hsapiens.v75 (2.99.0) [82], ensembldb (2.14.1) [83], and AnnotationFilter (1.14.0) [84] packages, where ensembldb *genes* function was applied to EnsDb.Hsapiens.v75 object with *AnnotationFilter* set up to ~ *gene_biotype* =  = *"protein_coding".* Only standard chromosomes were kept using GenomeInfoDb (1.26.7) [85] *keepStandardChromosomes* function. The output from the *computeMatrix* function was then plotted as a heatmap with deepTools *plotHeatmap* function with parameters—*kmeans 2 –whatToShow 'heatmap and colorbar'*.

Genes reported in cluster 1 were then passed to g:Profiler [86] for gene set enrichment analysis. Only KEGG, Reactome, WikiPathways and GO:BP terms were searched, as suggested by Reimand et al. [87]. Significant terms (padj < 0.05) were plotted as bar plots showing −log_10_(padj) values, except for GO:BP terms (due to a larger number of identified GO:BP terms), which were first processed by REVIGO [78] with default parameters, resulting network was further reorganized in Cytoscape (3.8.0) [79] and terms were manually classified into groups.

#### Analysis of transposable elements

A library of TEs (transposable elements) was obtained from RepeatMasker [88] library 20140131 for the hg19 genome assembly. Genomic coordinates for individual TE classes were extracted into BED files using R. Unambiguous classes such as RNA, DNA, or classes with a question mark in them were removed from further analysis. Overlaps with H3K9me3 regions were calculated using GenomicDistributions (1.5.5) [72] *calcExpectedPartitions* function with arguments *bpProportion* = *T*, and *genomeSize* set to sum of hg19 chromosome sizes, as instructed in the GenomicDistributions manual. Overlaps were then plotted with the *plotExpectedPartitions* function with added ggplot2 layer to show color intensity based on the overall size of overlap in number of base pairs.

To functionally annotate interesting TEs, intersections of all ERVs, LINE L1, SVA retrotransposons, and telomeric satellite DNAs with the top 10% significant, as well as all defined H3K9me3 regions, were obtained with the bedtools (v2.26.0) [61] *intersect* function. Intersections with the top 10% H3K9me3 regions were passed to GREAT [77], where intersections with all H3K9me3 regions were used as background. Maximum top 20 GO:BP terms, as displayed by GREAT, were then plotted with ggplot2 (3.3.6) [68] as bar plots showing negative log_10_ of FDR-corrected p-values.

Gene targets with significantly increased H3K27ac in 18-week-old children were obtained from Kupkova et al., 2021 [36] and compared to the gene targets of all ERVs within the top 10% significant H3K9me3 regions annotated by GREAT. Results were plotted as an upset plot using the *upset* function from the UpSetR package (1.4.0) [89]. Gene targets shared between ERVs and H3K27ac regions were visualized and functionally annotated with STRING [90].

#### Relationships between H3K9me3 in mothers and children

PCA applied to both sets of data was performed as described in the PCA section of Methods.

Results from differential analysis (negative log_2_(fold change) per unit change of child’s ΔHAZ score for both children and mothers) were plotted as box plots using ggplot2 (3.3.6) [68] and two-sided one-sample t-tests were used to determine any global unidirectional changes (H_0_: μ_log2FC_ = 0, H_1_: μ_log2FC_ ≠ 0, α = 0.05). To show direct comparisons of log_2_(fold changes) between children and mothers, H3K9me3 regions from mothers were overlapped with H3K9me3 regions from children with the bedtools (v2.26.0) [61] *intersect* function with parameter *-wao*, which returns the original coordinates for overlapping regions. Log_2_(fold changes) from the overlapping regions were then plotted as a scatter plot with ggplot2 and the Pearson’s correlation coefficient was calculated with the R *cor* function.

Correlation analysis between maternal and children’s samples was performed by first merging all maternal and child regions with bedtools *merge* followed by quantification of all maternal and child samples with bedtools *multicov*. A correlation matrix was then created by passing the count table to the R *cor* function with *method* = *“pearson”* and plotted as a heatmap using the *Heatmap* function from ComplexHeatmap (2.6.2) [91], where both rows and columns were clustered using average linkage.

To identify regions specific to mothers of stunted children/mothers of healthy children/stunted children/healthy children, all regions from all child and maternal datasets were first merged with the bedtools *merge* function to create a list of “master regions”. With the same function, regions of individuals belonging to a given category were merged, creating “category regions”. To compare the regions shared among or specific to categories, overlaps were found between “master regions” and individual “category regions” using bedtools intersect with the -wao parameter, which reports the original entries rather than the intersections. “Master regions” that were identified as overlapping with a given category were then passed to the *upset* function from UpSetR package (1.4.0) [89] to reveal the number of regions shared between a given combination of categories. Regions “shared in stunting” were defined as those “master regions” that had overlaps found with regions from mothers of stunted children and with regions from stunted children, but no other category, and analogically were defined regions “shared in health”. Summarization of the regions shared in health and shared in control individuals was performed using GenomicDistributions (1.5.5) [72], specifically, the c*alcFeatureDistRefTSS* function was used to compare the distances to the nearest TSS. The log_10_-transformed distances were then plotted with ggplot2 as density plots, as these provided an easier visual comparison for a smaller number of regions compared to the default histograms offered by GenomicDistributions. Cell-type specificity inference and distribution across genomic classes was calculated as described in the “Functional annotation of children’s H3K9me3 regions” section of Methods.

Gene set enrichment analysis of regions shared in stunting and regions shared in health was done by first assigning the regions to genes with GREAT [77] followed by passing the genes to g:Profiler [86] for identification of enriched biological terms (sources of biological terms were filtered to GO:BP, GO:MF (Gene Ontology: Molecular Function), KEGG, WikiPathways, Reactome). Significant terms were those with adjusted p-value < 0.05. Results with < 10 significantly enriched terms were plotted as bar plots showing −log_10_(padj) values. Larger sets of terms were passed as GEM files produced by g:Profiler to the EnrichmentMap app [92] in Cytoscape (3.8.0) [79], where terms were organized into networks followed by manual organization of related terms into clusters.

#### Integration with H3K4me3 and H3K27ac

Differential analysis results for H3K4me3 and H3K27ac data from 18-week-old and one-year-old children were obtained from Uchiyama et al. [35] and Kupkova et al. [36] respectively. These results show association of the H3K4me3 and H3K27ac peaks with a child’s ΔHAZ (birth:18wk) score for 18-week-old-children and ΔHAZ (birth:1yr) score for one-year-old children. Significantly affected regions were considered those with padj < 0.05. Regions were extracted from the result tables and converted to BED files. Overlaps with H3K9me3 regions from this study were found with the bedtools (v2.26.0) [61] *intersect* function with parameter *-wao* (to report original regions instead of just intersections). Proportions of overlaps were calculated as the ratio between an overlap and the size of a given region.

Gene set enrichment analysis of overlaps was done as described in the “Relationships between H3K9me3 in mothers and children” section above and histone mark/DBF enrichment analysis was performed as described in the “Functional annotation of children’s H3K9me3 regions” section above. The *userUniverse* in LOLA was set to overlaps between H3K9me3 and all upregulated H3K4me3 regions in one-year-old stunted children for H3K4me3 analysis, and to overlaps between H3K9me3 and all downregulated H3K27ac regions in one-year-old stunted children for H3K27ac analysis. For H3K27ac, only highly significant enrichments (padj < 0.001) were reported due to the large number of enriched histone marks and DBFs.

#### Additional tools used

Tidyverse package (1.3.1) [93] was used to process data in R. Figures were assembled with Inkscape (1.0.2) [94], Illustrations were created with BioRender [95], and icons were obtained from the Noun Project [96].

### Supplementary Information

**Additional file 1**. Supplementary figures (S1-S14), Table S1-2.

**Additional file 2: Table S3**. Differential analysis results infants.

**Additional file 3: Table S4**. Differential analysis results mothers.

## Data Availability

All raw sequencing FASTQ files generated in this study are available in dbGaP repository, phs001073.v3.p1, https://www.ncbi.nlm.nih.gov/gap/. DbGaP identifiers for the samples used in this study are listed in Additional file 1:Table S1-2.

## Abbreviations

ChIP-seq: Chromatin immunoprecipitation followed by sequencing
DBF: DNA binding factor
EED: Environmental enteric dysfunction
ERVs: Endogenous retroviruses
GOBP: Gene ontology: biological process
GOMF: Gene ontology: molecular function
H3K4me1: Histone H3 lysine 4 monomethylation
H3K4me3: Histone H3 lysine 4 trimethylation
H3K9me3: Histone H3 lysine 9 trimethylation
H3K27ac: Histone H3 lysine 27 acetylation
H3K36me3: Histone H3 lysine 36 trimethylation
HAZ: Height-for-age z
IFN-γ: Interferon gamma
IL8: Interleukin 8
LINE L1: Long interspersed nuclear elements-L1
NF: Normalization factor
PBMCs: Peripheral blood mononuclear cells
PC1: First principal component
PC2: Second principal component
PCA: Principal component analysis
PROVIDE: Performance of rotavirus and oral polio vaccines in developing countries (study)
PU.1: Hematopoietic transcription factor PU.1 (SPI1)
SiH: Regions shared in health
SiS: Regions shared in stunting
SPI1: Hematopoietic transcription factor PU.1
SVA: SINE-R-VNTR-Alu
TEs: Transposable elements
TF: Transcription factor
TSS: Transcription start site
TGFβ: Transforming growth factor-β
WAZ: Weight-for-age z
ZAP: Zinc-finger antiviral protein
