# Supplementary material for: Globally elevated levels of histone H3 lysine 9 trimethylation in early infancy are associated with poor growth trajectory in Bangladeshi children
Source: Clin Epigenetics. 2023 Aug 11;15:129. doi: 10.1186/s13148-023-01548-z (PMC10422758; doi:10.1186/s13148-023-01548-z)
Supplement: Supplementary file 1 — Additional file 1. Supplementary figures (S1-S14), Table S1-2. [file 13148_2023_1548_MOESM1_ESM.docx]

Supplementary material

Globally elevated levels of histone H3 lysine 9 trimethylation in early infancy associated with poor growth trajectory in Bangladeshi children

Kristyna Kupkova, Savera J. Shetty, Marilyn G. Pray-Grant, Patrick A. Grant, Rashidul Haque, William A. Petri Jr., and David T. Auble^🖂^

**^🖂^** Correspondence: [auble@virginia.edu](mailto:auble@virginia.edu).

Table of Contents

[Supplementary figures 2](#_Toc140407091)

[Fig. S1. HAZ and ΔHAZ scores of children used in this study vs. all children from PROVIDE cohort. 2](#_Toc140407092)

[Fig. S2. Sex representation in all the PROVIDE children and children whose samples were used in this study. 3](#_Toc140407093)

[Fig. S3. Sex separated average H3K9me3 within H3K9me3 peaks. 4](#_Toc140407094)

[Fig. S4. Global H3K9me3 and H3 measurements. 4](#_Toc140407095)

[Fig. S5. Use of *Drosophila melanogaster* spike-in normalization to uncover unidirectional global changes in H3K9me3 levels. 5](#_Toc140407096)

[Fig. S6. Summary of all H3K9me3 regions and the top 10% significantly misregulated regions relative to ΔHAZ (birth:1yr) score. 7](#_Toc140407097)

[Fig. S7. Gene set enrichment of the genes associated with the 10% significant H3K9me3 regions. 9](#_Toc140407098)

[Fig. S8. H3K9me3 at gene sites. 10](#_Toc140407099)

[Fig. S9. Enrichment of transposable elements in misregulated H3K9me3 regions and their potential function. 11](#_Toc140407100)

[Fig. S10. Relationship between H3K9me3 profiles of mothers and 18-week-old infants. 12](#_Toc140407101)

[Fig. S11. Annotation of H3K9me3 regions shared in health vs. shared in stunting between mothers and children. 14](#_Toc140407102)

[Fig. S12. H3K4me3 and H3K27ac results in stunting. 16](#_Toc140407103)

[Fig. S13. Proportional overlaps of H3K4me3 and H3K27ac across different ages with H3K9me3 at 18 weeks. 17](#_Toc140407104)

[Fig. S14. Annotation of significantly upregulated H3K4me3 (top panels) and downregulated H3K27ac (bottom panels) regions in 1-year-old stunted children that overlap H3K9me3 regions in 18-week-old children. 18](#_Toc140407105)

[Supplementary tables 19](#_Toc140407106)

[Table S1. Preprocessing statistics. 19](#_Toc140407107)

[Table S2. Sample identifiers and associated measurements. 20](#_Toc140407108)

[Supplementary text 21](#_Toc140407109)

[Supplementary references 21](#_Toc140407110)

# Supplementary figures


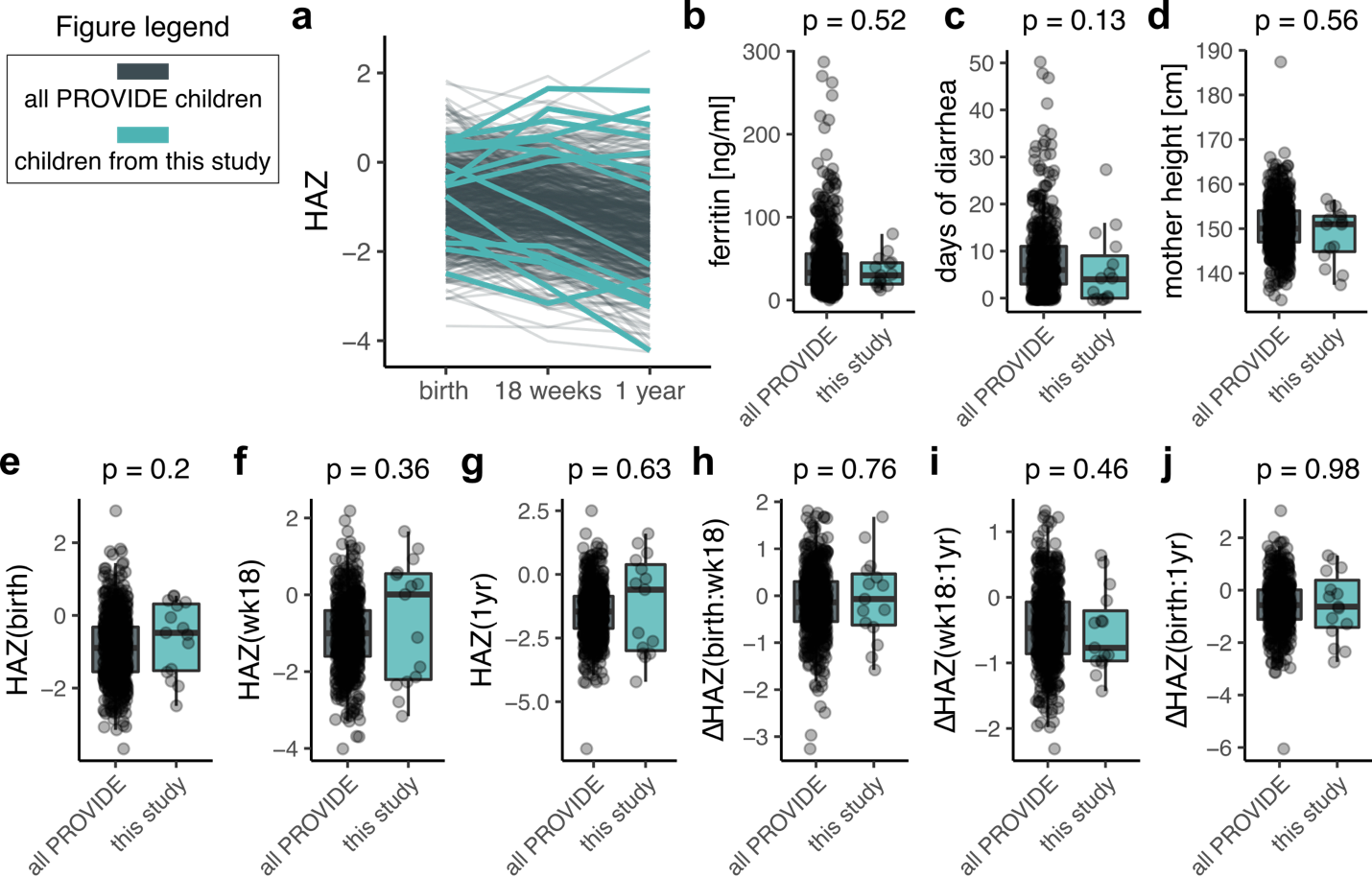


Fig. S1. HAZ and ΔHAZ scores of children used in this study vs. all children from PROVIDE cohort.

(a) Growth trajectories within the first year of life of children selected for this study (n = 15, green) and all children from the PROVIDE study (n = 700, dark grey). Each line represents one child and connects HAZ scores of the child at birth, 18 weeks and one year. (b-j) Box plots showing distribution of measurements from all of the children enrolled in the PROVIDE study (dark grey) and children selected for this study (green). The following measurements are shown: (b) ferritin levels, (c) days of diarrhea within the first 18 weeks, (d) maternal height, (e) HAZ at birth, (f) HAZ at 18 weeks, (g) HAZ at one year, (h) ΔHAZ between birth and 18 weeks, (i) ΔHAZ between 18 weeks and one year, (j) ΔHAZ between birth and one year. Shown in box plots are the p-values of two-sided Mann-Whitney U test, significant p < 0.05. Boxplot horizontal line indicates median value, the boxes show interquartile range (IQR) between 25^th^ percentile (Q1) and 75^th^ percentile (Q3), and the whiskers show minimum value excluding outliers (Q1 – 1.5*IQR), and maximum value excluding outliers (Q3 + 1.5 *IQR). Overlaid over box plots are points indicating actual values.


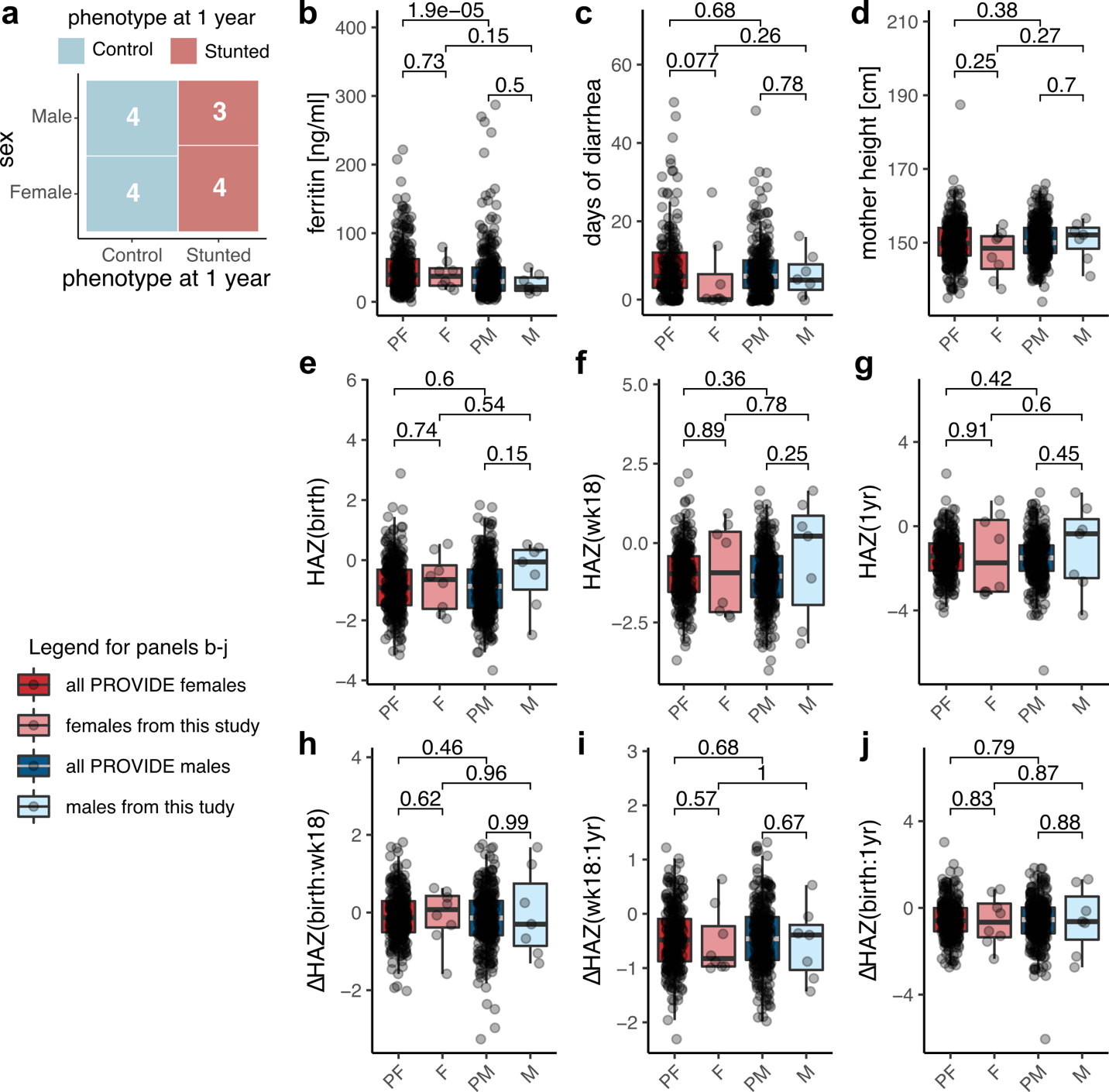


Fig. S2. Sex representation in all the PROVIDE children and children whose samples were used in this study.

(a) Mosaic plot showing the number of control vs. stunted (x-axis) males and females (y-axis) whose samples were used in this study. (b-j) Box plots showing distribution of measurements from all of the PROVIDE female children (PF), female children from this study (F), all of the male PROVIDE children (PM), and male children from this study (M). Shown are the following measurements: (b) ferritin levels, (c) days of diarrhea within the first 18 weeks, (d) maternal height, (e) HAZ at birth, (f) HAZ at 18 weeks, (g) HAZ at one year, (h) ΔHAZ between birth and 18 weeks, (i) ΔHAZ between 18 weeks and one year, (j) ΔHAZ between birth and one year. Shown in box plots are p-values of two-sided Mann-Whitney U test, significant p < 0.05. Boxplot horizontal line indicates median value, the boxes show interquartile range (IQR) between 25^th^ percentile (Q1) and 75^th^ percentile (Q3), and whiskers show minimum value excluding outliers (Q1 – 1.5*IQR), and maximum value excluding outliers (Q3 + 1.5 *IQR). Overlaid over box plots are points indicating actual values.


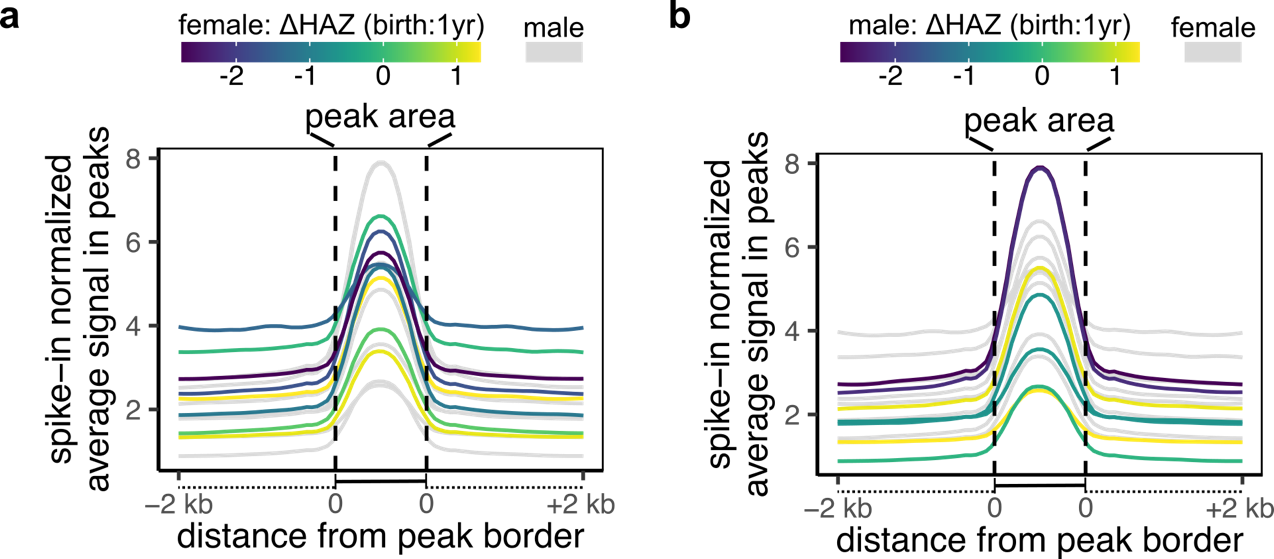


Fig. S3. Sex separated average H3K9me3 within H3K9me3 peaks.

(a,b) Figures same as Fig. 2a in the main text but highlighting female (a) and male (b) samples.


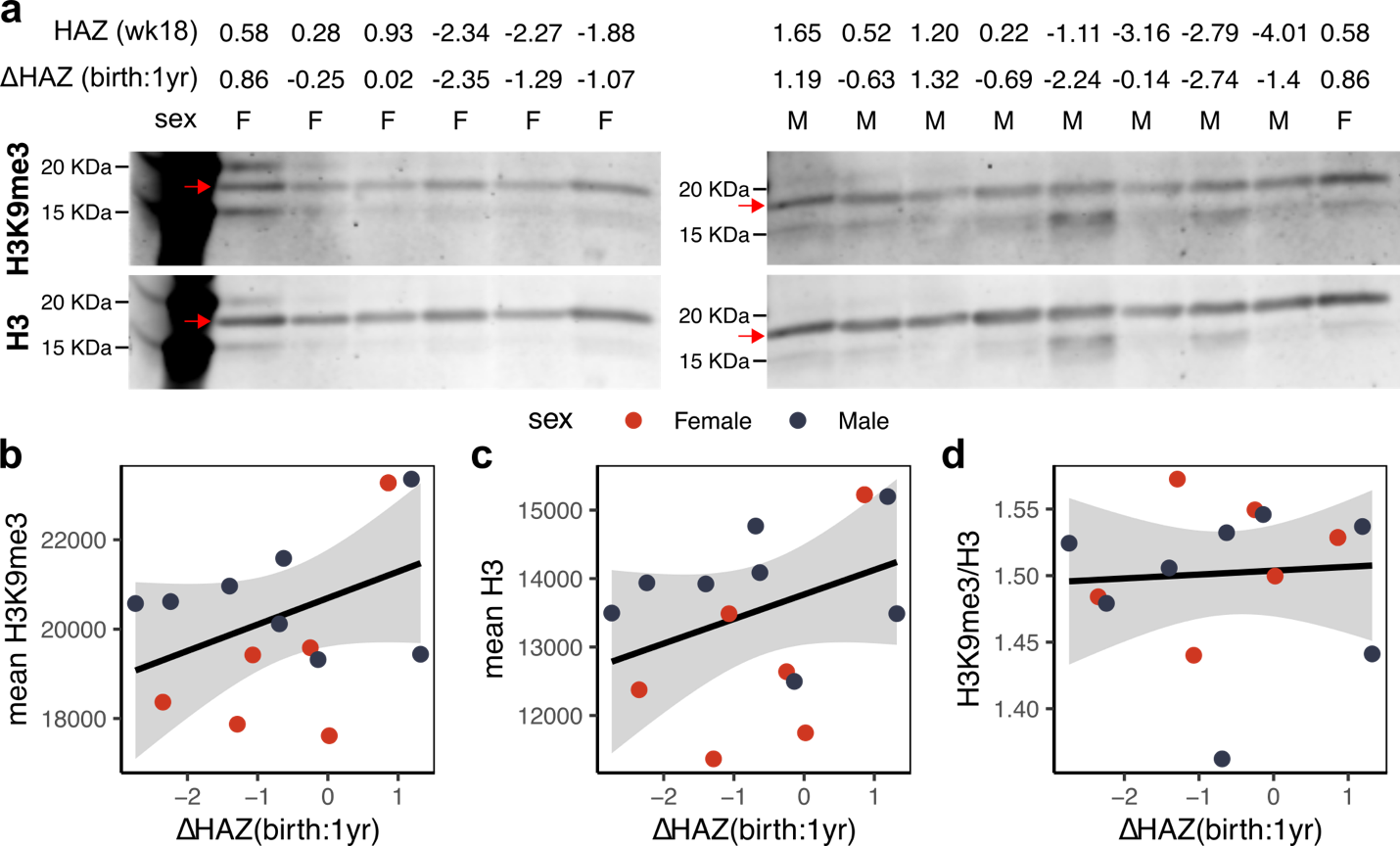


Fig. S4. Global H3K9me3 and H3 measurements.

Total H3K9me3 and H3 measured by Western blotting. While a shorter species of H3 was detectable in some samples, the shorter form retained the H3K9me3 modification suggesting no significant loss in H3K9me3 signal due to N-terminal clipping. The red arrows indicate the full-length band. The most left and most right samples are technical replicates (sex = F, HAZ (wk18) = 0.58, ΔHAZ (birth:1yr) = 0.86). (b) Quantified H3K9me3 levels. Linear model controlling for sex differences: p (ΔHAZ (birth:1yr)) = 0.11, adjusted R^2^ = 0.11, DF = 12. (c) Quantified H3 levels. Linear model controlling for sex differences: p (ΔHAZ (birth:1yr)) = 0.13, adjusted R^2^ = 0.14, DF = 12. (d) H3K9me3 to H3 ratios. Linear model controlling for sex differences: p (ΔHAZ (birth:1yr)) = 0.85, adjusted R^2^ = -0.1, DF = 12. P-value < 0.05 is considered significant. The quantified levels of samples from (a, left) were normalized using the technical replicates. Note that there is no significant relationship between total H3, total H3K9me3 nor the H3K9me3/H3 ratio and ∆HAZ (birth:1yr).


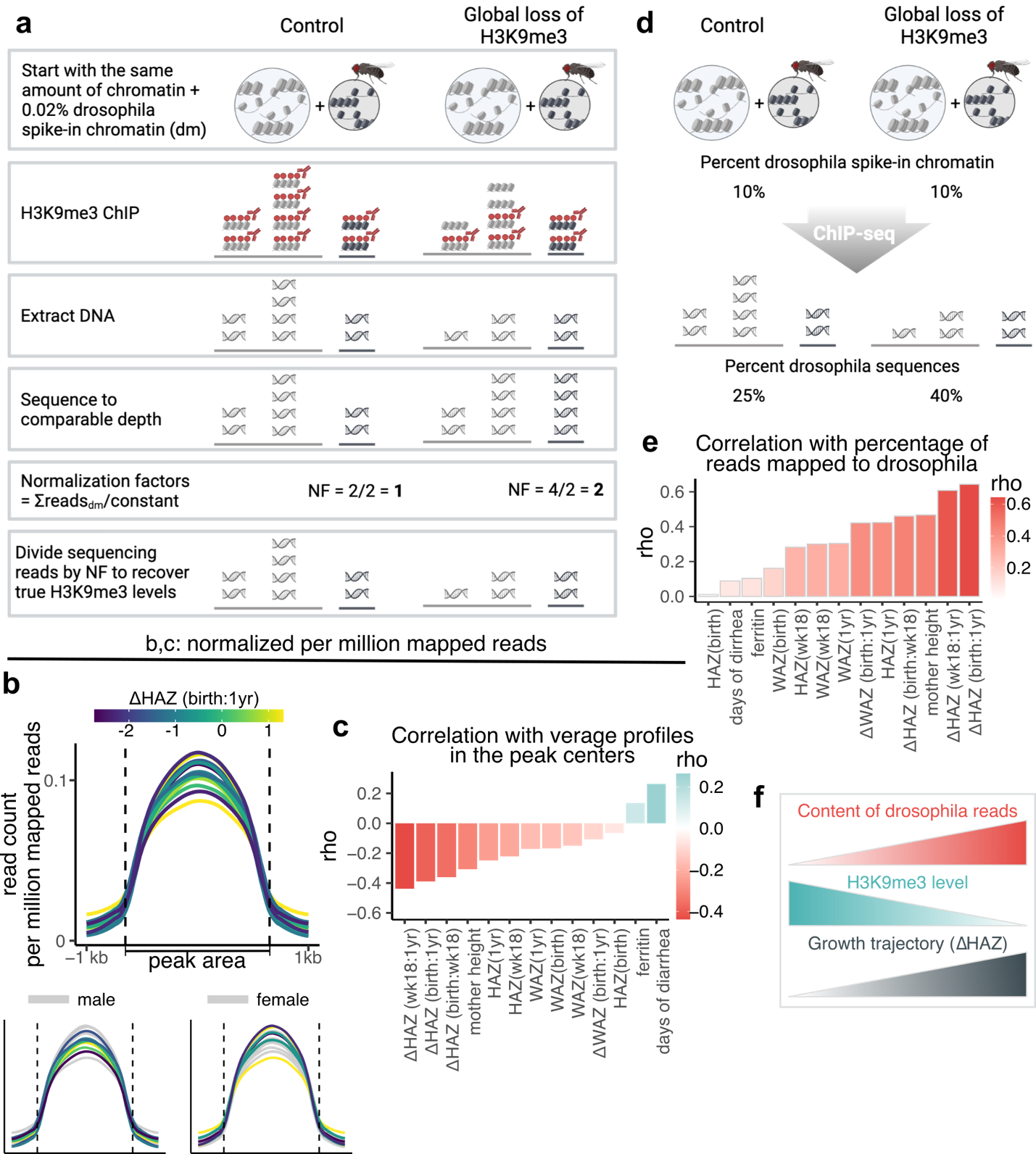


Fig. S5. Use of *Drosophila melanogaster* spike-in normalization to uncover unidirectional global changes in H3K9me3 levels.

(a) Illustration showing how use of spike-in chromatin is used to normalize samples to uncover true levels of ChIP-seq targets. (b) Top: Average H3K9me3 profiles within peaks and 2 kb regions surrounding peaks. Each line represents an average profile of a child color coded by the child’s ΔHAZ (birth**:**1yr) score. The average profiles were normalized to million mapped reads. Bottom panels highlight sex differences in the top panel. Bottom left panels show color coded females and males in gray, bottom right shows color coded males and females in gray. (d) Illustration showing that increased percentage of spike-in reads within a sample is reflective of global loss of ChIP-seq target. Starting with the same amount of chromatin material should result in the same percentage of spike-in reads in the sample under normal circumstances, however, when less material is pulled down with ChIP, it leads to an increased proportion of spike-in reads. (e) Bar plot showing Spearman correlation coefficients between percentage of spike-in reads and all available measurements reflective of child’s health status. (f) Summary illustration. Increased percentage of spike-in reads negatively correlates with global H3K9me3 levels and positively correlates with ΔHAZ (birth:1yr) score. Therefore, globally increased H3K9me3 levels are associated with low ΔHAZ (birth:1yr) score indicative of poor growth change.


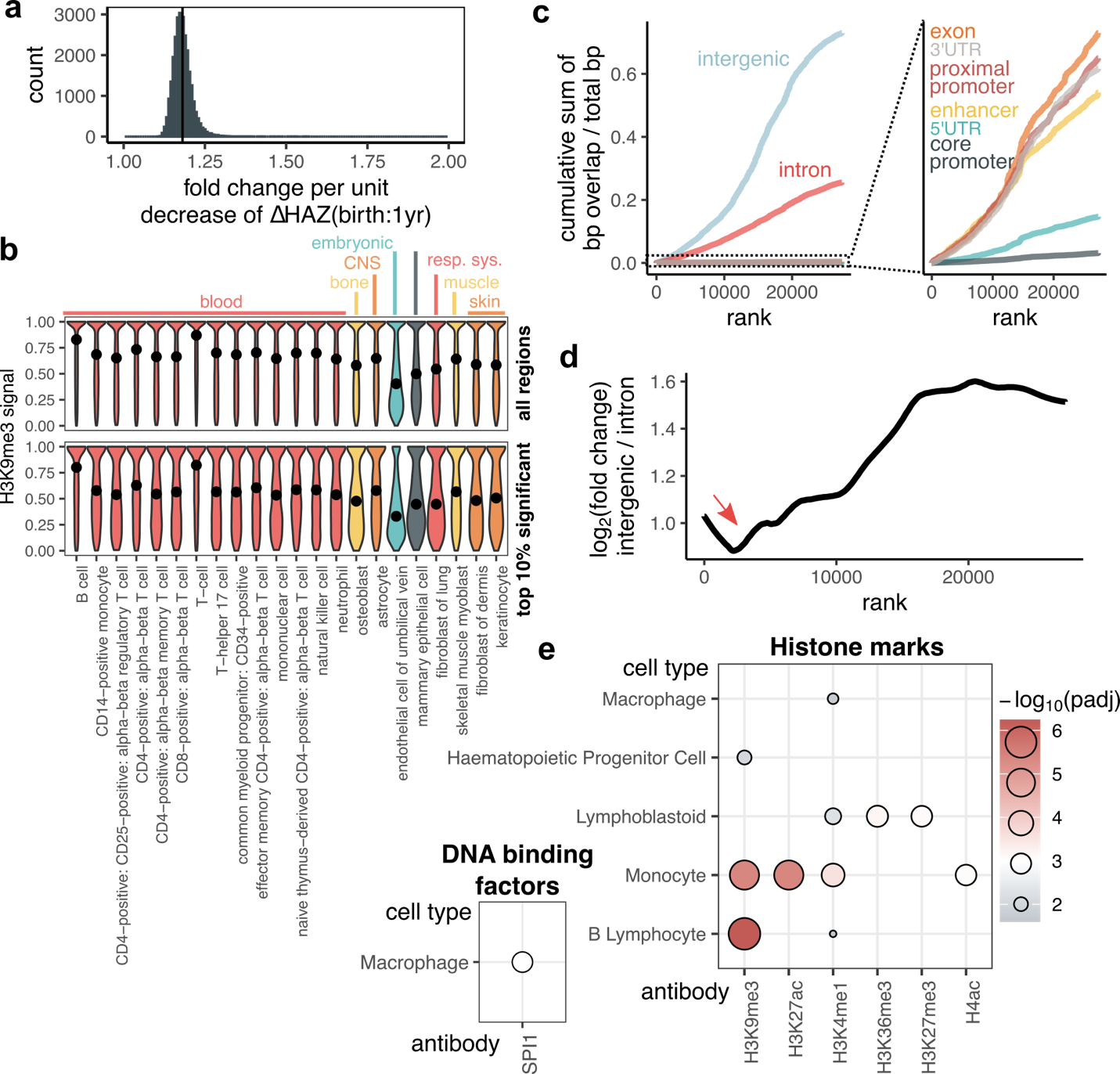


Fig. S6. Summary of all H3K9me3 regions and the top 10% significantly misregulated regions relative to ΔHAZ (birth:1yr) score.

(a) Histogram of fold changes per unit decrease in ΔHAZ (birth:1yr) score. The vertical line indicates the mean value: 1.18. (b) Violin plots showing distribution of normalized H3K9me3 signal values within regions of interest across different cell types. And increased values within a certain cell type are potentially indicative of cell-type specificity of the regions. Compared here are H3K9me3 signal distributions within all H3K9me3 identified regions in this study (top panel) vs. within the top 10% significantly affected H3K9me3 regions relative to ΔHAZ (birth:1yr) score. The black dots indicate median values. The top 10% significant regions lie within regions with overall lower normalized H3K9me3 regions (Mann Whitney U test, H_0_: the distributions are equal, H_1_: the values within the top 10% significant regions are lower, p < 0.5 for all cell types). Medians are highly correlated (r = 0.97) suggesting no preference of the top 10% significant regions towards a particular cell type. (c) Cumulative distribution of H3K9me3 regions across genomic classes. H3K9me3 regions are ranked based on adjusted p-value followed by p-value (to distinguish ranks of regions with the same adjusted p-value). Calculated are the number of bp (base pairs) for each region that overlap a given genome class. Plotted is the cumulative sum of the bp overlaps normalized to the total number of bp within the H3K9me3 regions of interest. Panel on the right shows zoom-in at genomic classes with low overlap with H3K9me3 regions. (d) Log_2_(fold change) of cumulative distributions between intergenic and intronic regions from (c). Red arrow indicates a slight drop in log_2_(fold change) within the top 10% ranked H3K9me3 regions indicative of slightly higher enrichment of introns within these regions. (e) Enrichment of DBFs (DNA binding factors, left) and histone marks (right) within the top 10% most significant H3K9me3 regions. The color and the size of the dots are reflective of -log_10_(padj) for a given DBF- or histone mark-cell type combination.


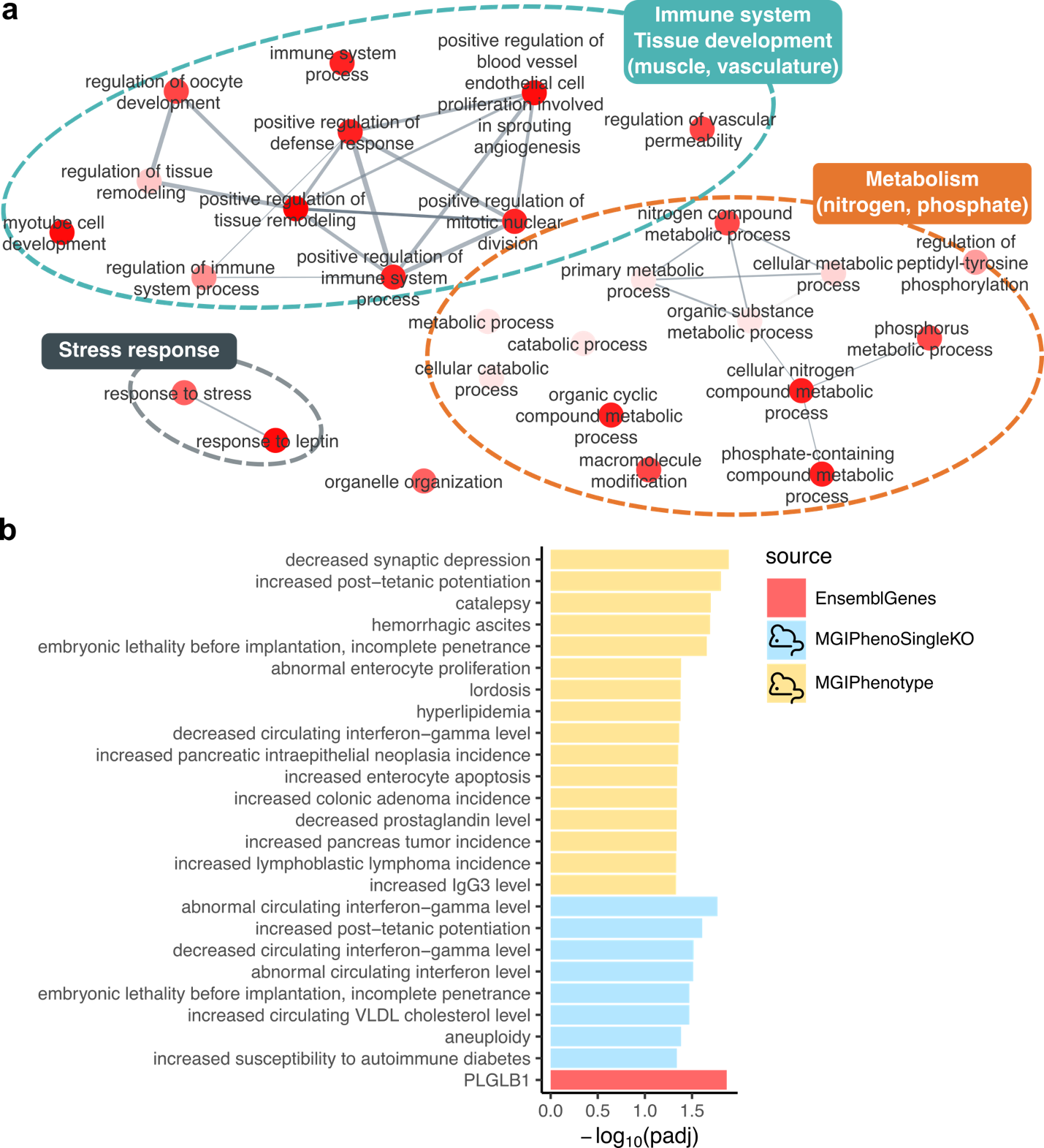


Fig. S7. Gene set enrichment of the genes associated with the 10% significant H3K9me3 regions.

(a) Network showing GO:BP terms associated with the top 10% significant H3K9me3 regions. GO:BP were manually grouped into clusters indicated. The saturation of red indicates significance of the gene set enrichment (bright red – highly significant). (b) Terms with significant gene set enrichment other than GO:BP. The mouse icon in the legend indicates gene sets obtained from mouse studies.


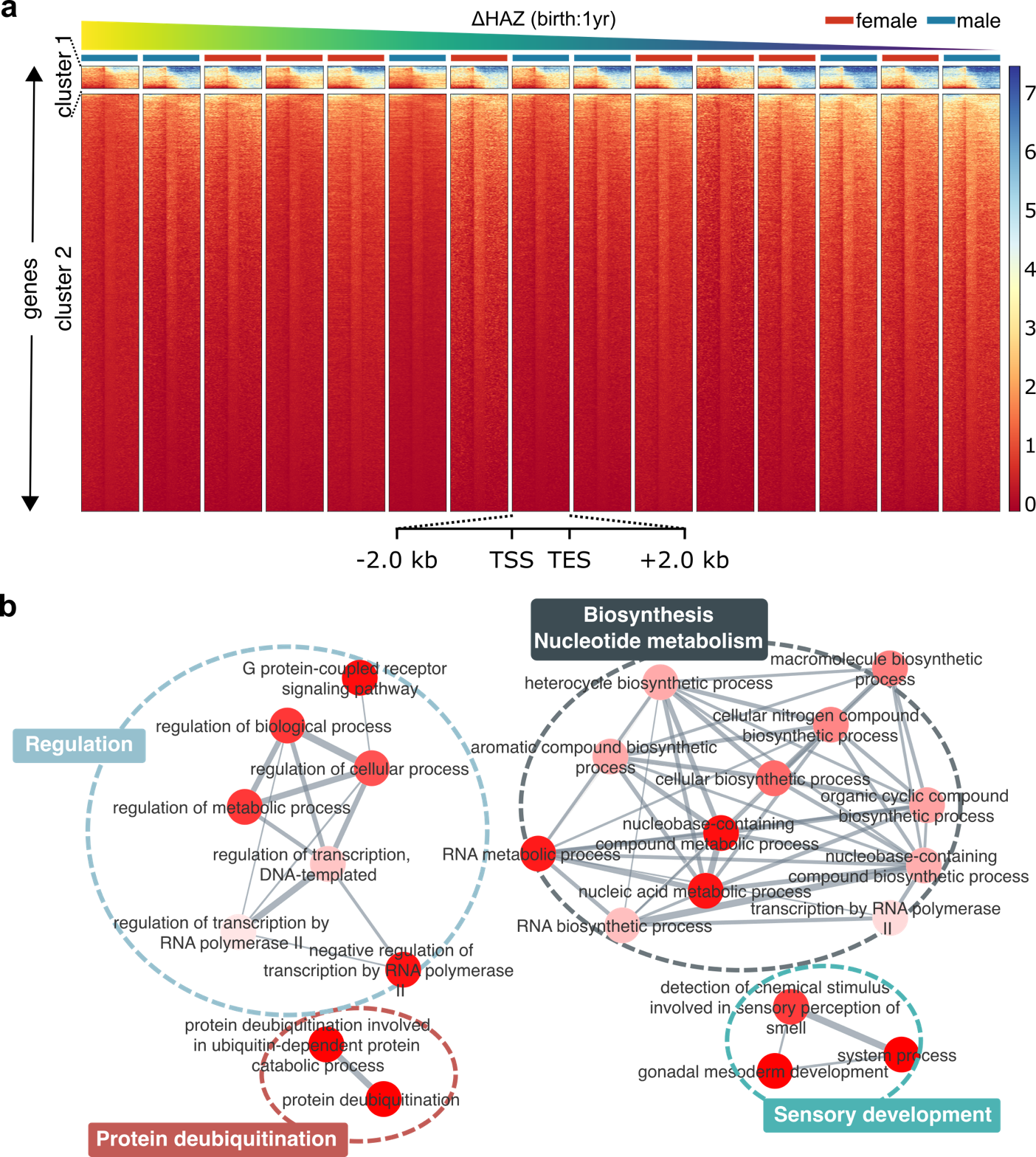


Fig. S8. H3K9me3 at gene sites.

(a) Heatmaps showing normalized H3K9me3 coverage over genes and ±2 kb surrounding regions. Shown are two gene clusters generated with k-means clustering. Heatmaps are ordered based on child’s ΔHAZ (birth:1yr) score from high to low (left to right). (b) GO:BP terms with significant gene set enrichment of genes from cluster 1 in panel (a). Individual terms were manually grouped into clusters. The saturation of red indicates significance of the gene set enrichment (bright red – highly significant).


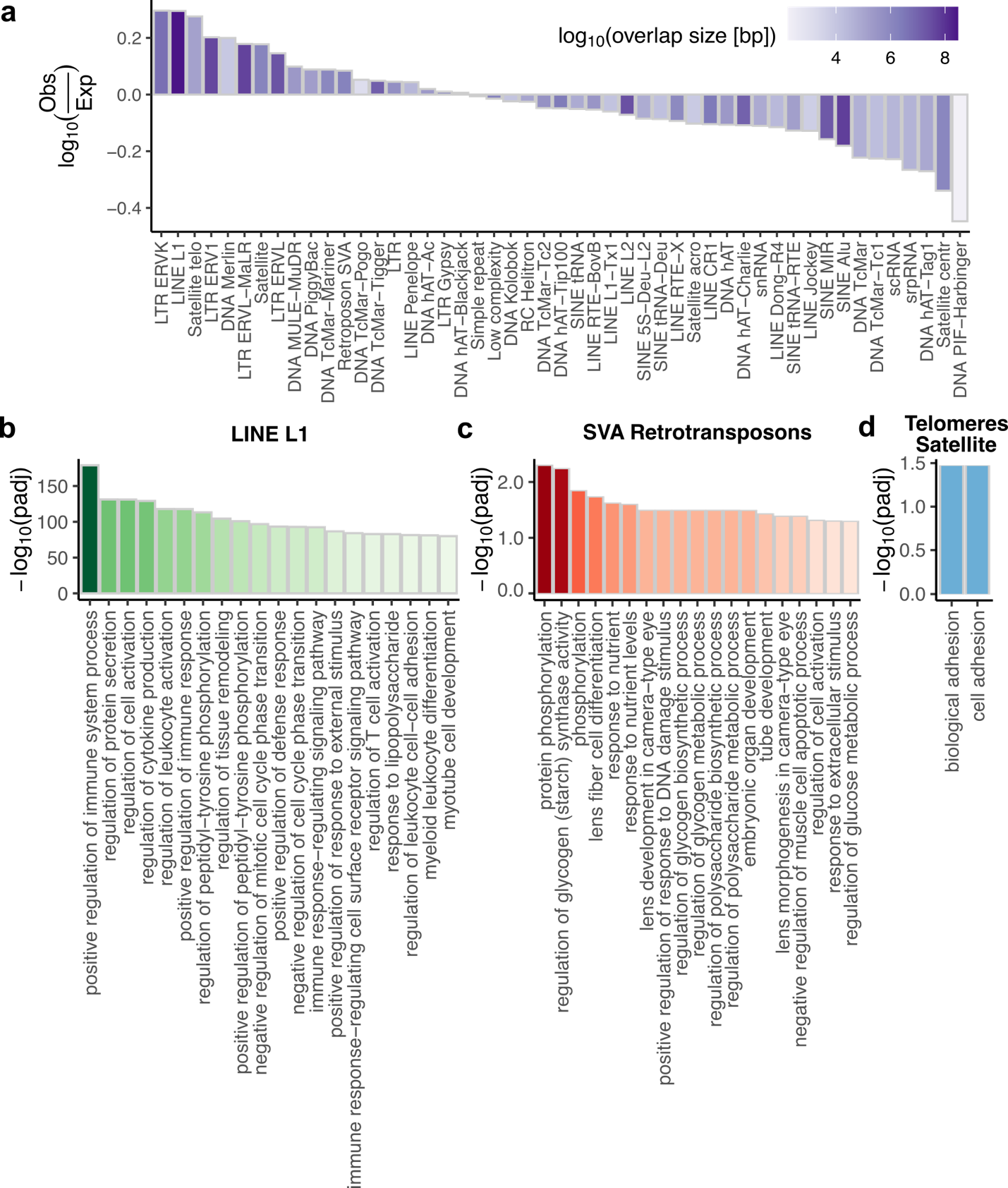


Fig. S9. Enrichment of transposable elements in misregulated H3K9me3 regions and their potential function.

(a) Ratio of observed over expected overlaps of TEs with top 10% significantly misregulated H3K9me3 regions with ΔHAZ (birth:1yr) score. Positive values indicate overlaps higher than expected. The color shows the total overlap size in base-pairs for a given TE class. (b)-(d) Top 20 GO:BP terms, as displayed by GREAT, associated with LINE L1 (b), SVA retrotransposons (c), and satellite DNA in telomeres (d) within the top 10% significantly affected H3K9me3 regions. Plotted are negative log_10_ FDR-corrected p-values (padj). Padj < 0.05 are considered significant.


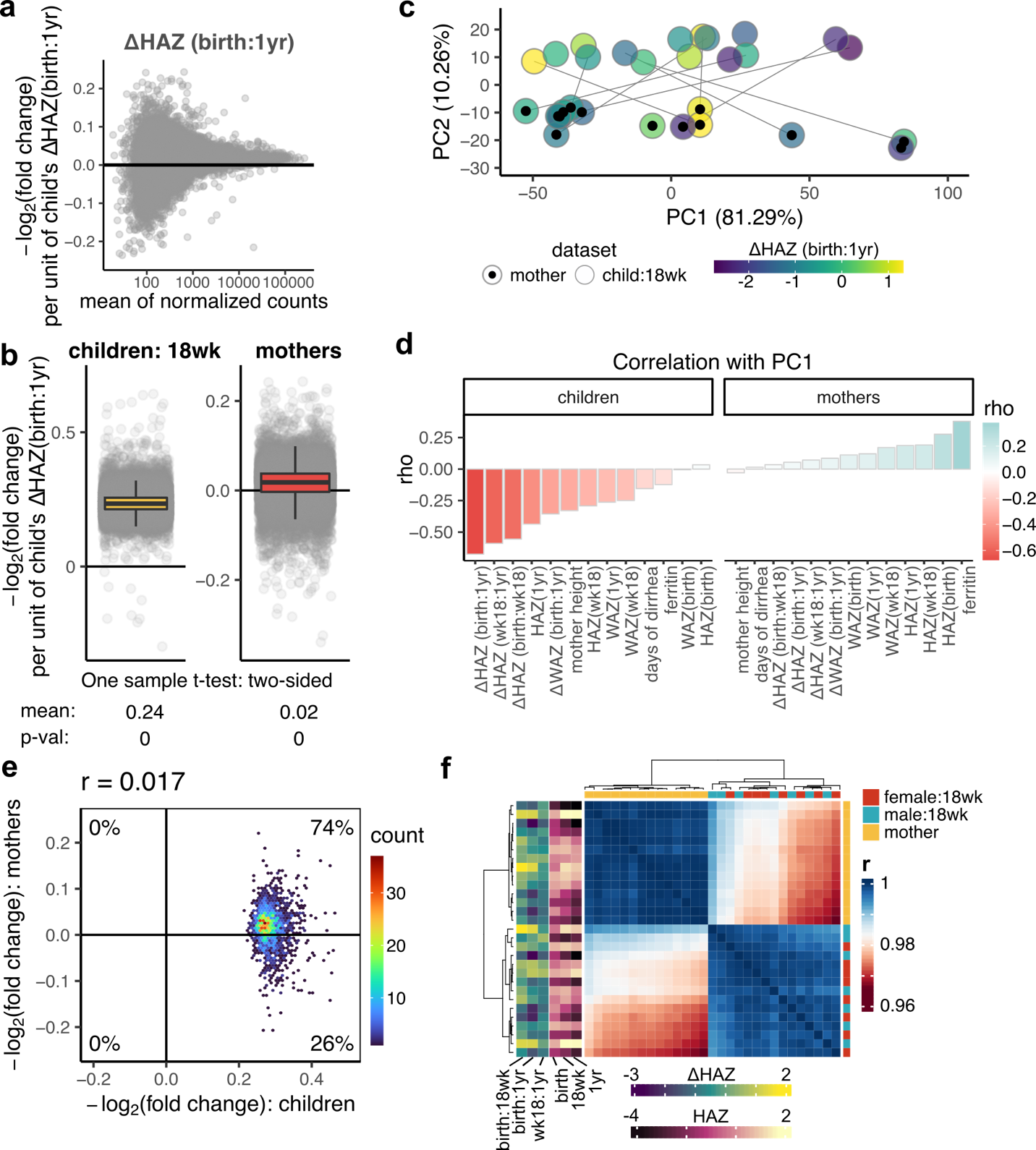


Fig. S10. Relationship between H3K9me3 profiles of mothers and 18-week-old infants.

(a) MA-plot shows changes of maternal H3K9me3 levels with negative unit changes of child’s ΔHAZ (birth:1yr) score. Each point is and identified H3K9me3 region, x-axis shows the mean normalized H3K9me3 coverage and y-axis shows -log_2_(fold change) of H3K9me3 coverage per unit increase of child’s ΔHAZ (birth:1yr) score. Regions whose coverage increases in association with negative growth trajectory are above x-axis. Lack of points highlighted in other color indicates that none of the H3K9me3 regions are significantly associated with child’s ΔHAZ (birth:1yr) score. Significance threshold: padj < 0.05. (b) Box plots showing negative log_2_(fold changes) of H3K9me3 levels per unit change of child’s ΔHAZ (birth:1yr) for dataset from 18-week-old children (left) and from mothers (right). Two-sided t-test p-values under each plot indicates significant difference in overall H3K9me3 levels, which are in both cases upregulated with negative change of child’s ΔHAZ (birth:1yr) score. Boxplot horizontal line indicates median value, the boxes show interquartile range (IQR) between 25^th^ percentile (Q1) and 75^th^ percentile (Q3), and whiskers show minimum value excluding outliers (Q1 – 1.5*IQR), and maximum value excluding outliers (Q3 + 1.5 *IQR). Overlaid over box plots are points indicating actual values. (c) PCA plot of normalized H3K9me3 counts from mothers and children. Each point represents an individual and is color coded based on child’s ΔHAZ (birth:1yr) score. (d) Spearman’s correlation coefficients between PC1 from panel (c) and all available measurements. Left panel shows correlations for children and right panel for mothers. (e,f) Panels as in Fig. 5b and Fig. 5d in the main text, respectively, however, highlighting relationships within the top 10% significantly misregulated regions in children.


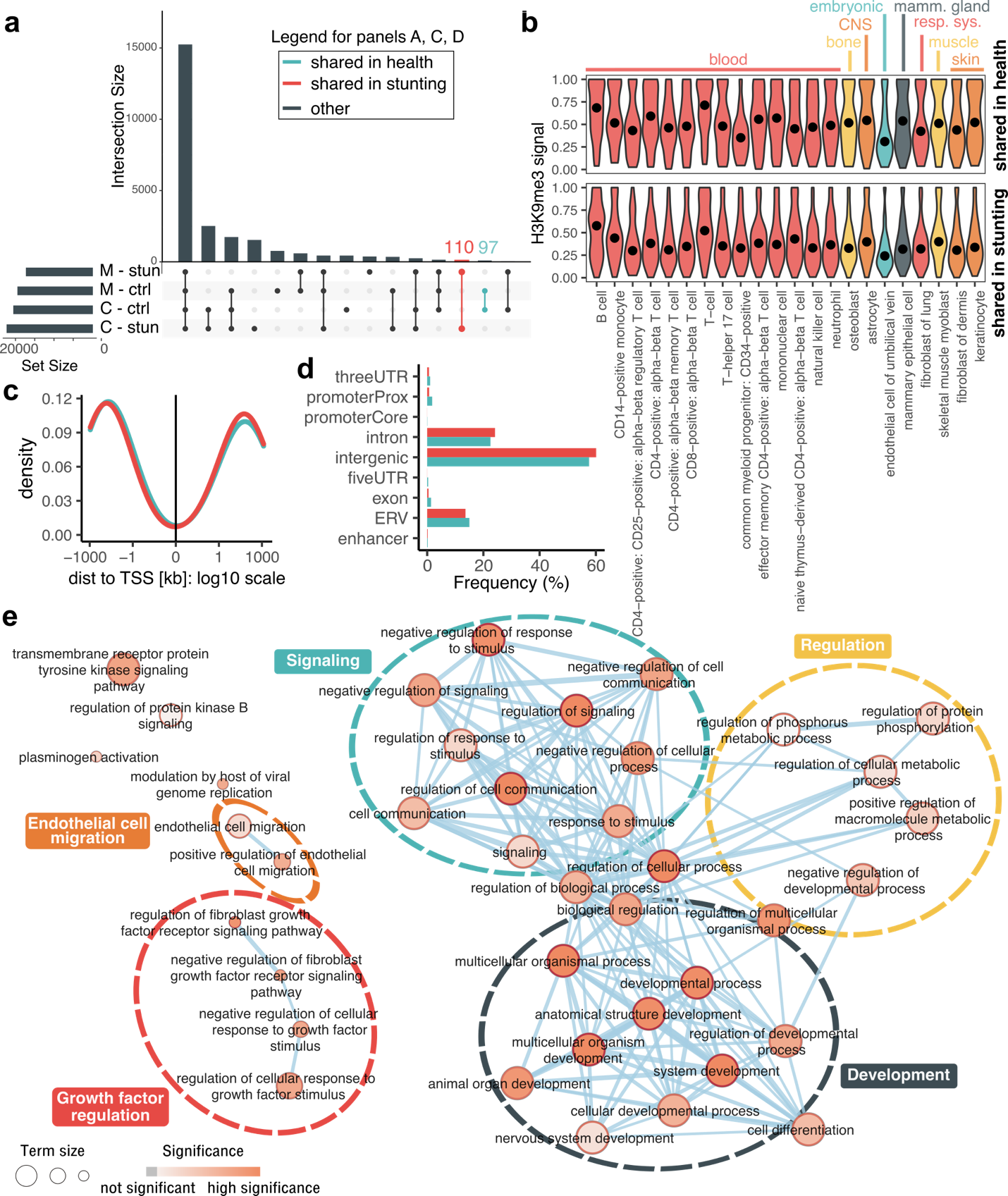


Fig. S11. Annotation of H3K9me3 regions shared in health vs. shared in stunting between mothers and children.

(a) Upset plot showing number of regions shared between specified categories of individuals. M-stun: mothers of stunted children, M-ctrl: mothers of control children, C-ctrl: control children, C-stun: stunted children. (b) Violin plots showing distribution of normalized H3K9me3 signal values within regions of interest across different cell types. And increased values within a certain cell type are potentially indicative of cell-type specificity of the regions. Compared here are H3K9me3 signal distributions within regions shared in health (top panel) vs. regions shared in stunting (bottom panel). The black dots indicate median values. (c) Density plot showing distances to the nearest TSS of regions shared in health (green) vs. regions shared in stunting (red). (d) Overlaps of regions shared in health and shared in stunting with genomic classes. (e) Gene set enrichment of genes in proximity to regions shared in health. Each node represents a biological term, the node size corresponds to the term size, and the color is based on adjusted p-value of the enrichment. The edge width is based on the number of genes shared between connected terms.


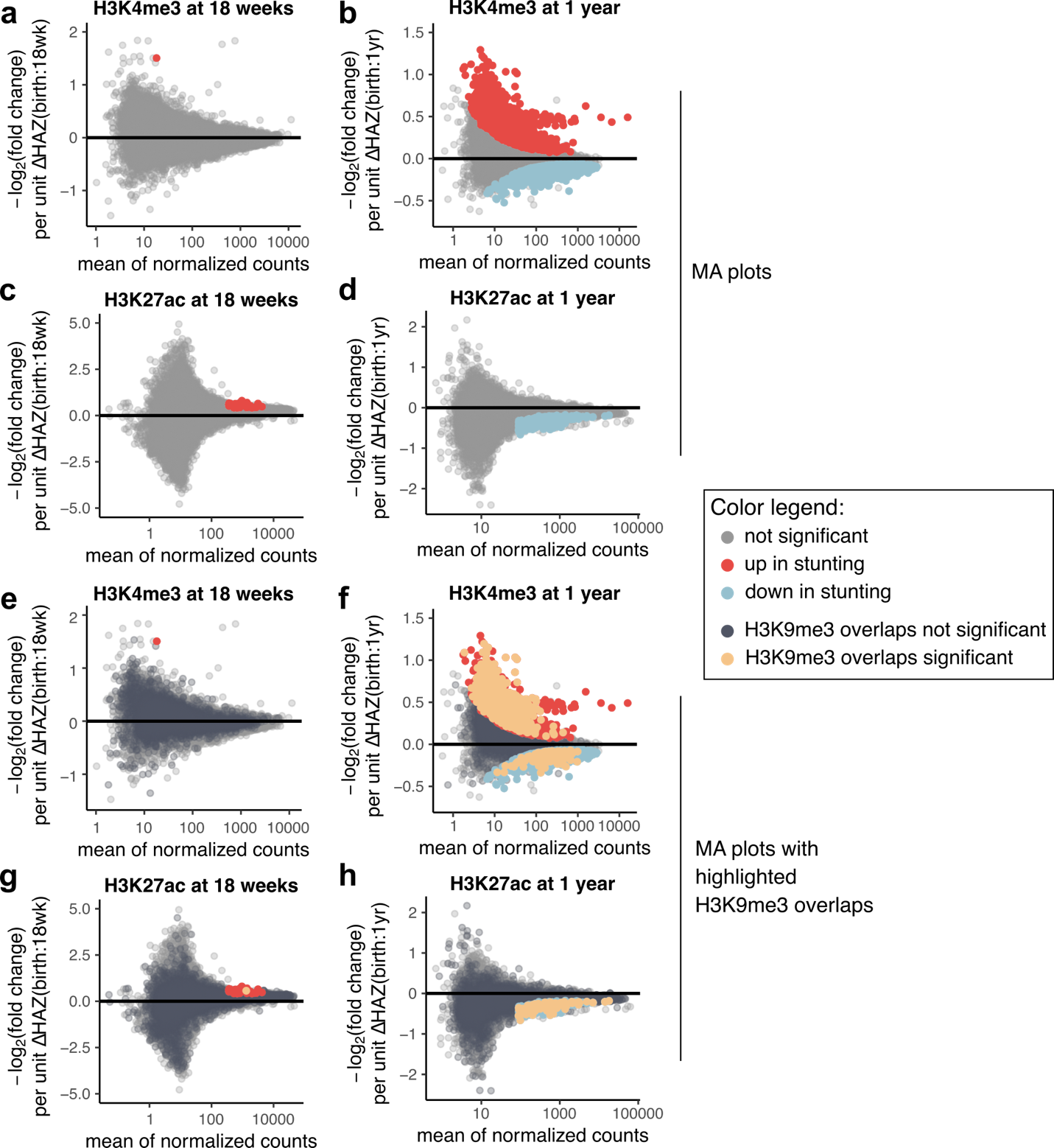


Fig. S12. H3K4me3 and H3K27ac results in stunting.

(a-d) MA-plots showing negative log_2_(fold changes) in levels of a given histone mark at a given age, as indicated in the panel title, per unit change of ΔHAZ score between birth and a corresponding age (y-axis). Values on x-axis represent mean normalized read coverage of a given region. Regions significantly upregulated with low ΔHAZ score are shown in red, and regions downregulated with low ΔHAZ score are shown in blue. Significant values are those with padj < 0.05. (e-h) Same as panels (a-d), just highlighting, which regions overlap H3K9me3 regions from this study. Overlaps of H3K4me3 or H3K27ac regions that are not significantly associated with ΔHAZ score are shown in dark grey, those regions that are significantly associated with ΔHAZ score and overlap H3K9me3 regions are shown in yellow.


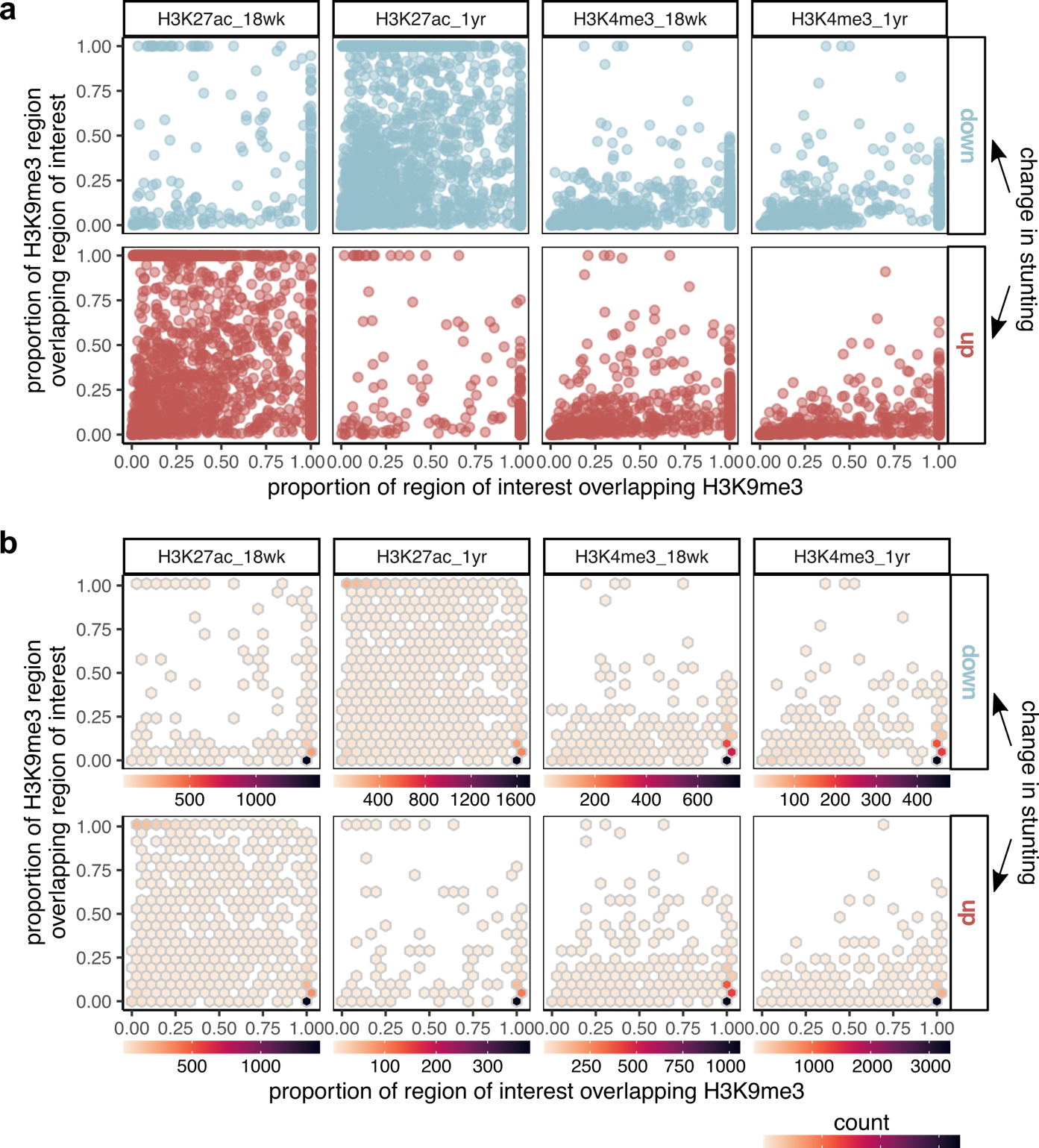


Fig. S13. Proportional overlaps of H3K4me3 and H3K27ac across different ages with H3K9me3 at 18 weeks.

(a) Proportional overlap o H3K9me3 regions (y-axis) vs. proportion of H3K27ac or H3K4me3 region that overlaps H3K9me3 region (x-axis) as illustrated in Fig. 5D in the main manuscript. H3K4me3 and H3K27ac come from 18-week-old (18wk) and one-year-old children (1yr) as indicated in the panel title. The top panels show regions that are upregulated in stunted children (upregulated with decreasing ΔHAZ score) and bottom panels regions that are downregulated in stunted children (downregulated with decreasing ΔHAZ score). (b) Panels as in (a) color coded based on number of points falling into a given bin.


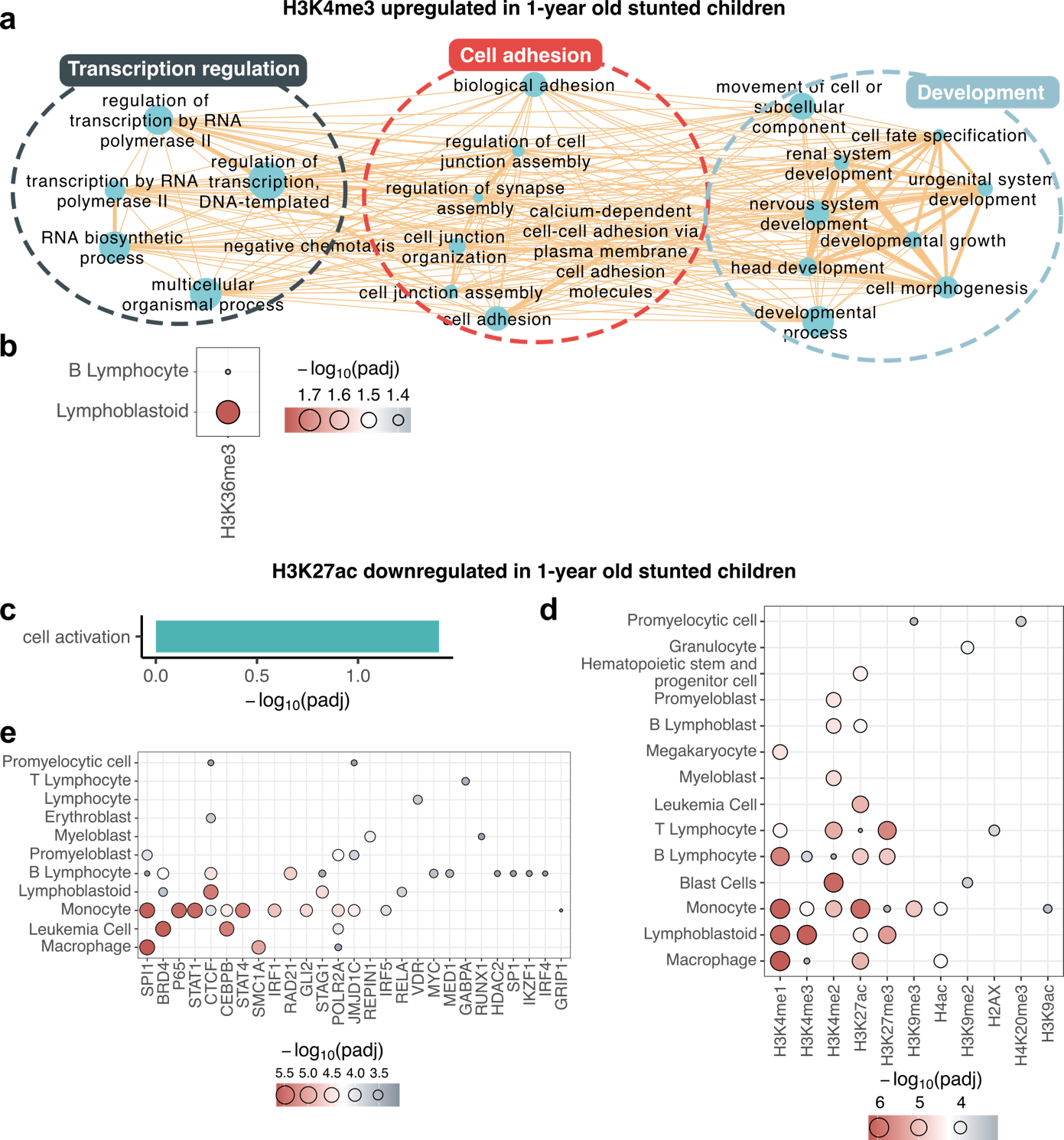


Fig. S14. Annotation of significantly upregulated H3K4me3 (top panels) and downregulated H3K27ac (bottom panels) regions in 1-year-old stunted children that overlap H3K9me3 regions in 18-week-old children.

(a-b) Annotation of H3K9me3 overlaps with significantly upregulated H3K4me3 regions in 1-year-old stunted children. (a) Gene set enrichment of genes associated with the overlaps. Each node is a biological term (source - GO:BP), size of the node corresponds to the size of the term and the width of the edge is indicative of number of genes shared between the connected nodes. Shown are terms with significant enrichments based on adjusted p-value < 0.05. Terms were manually grouped into highlighted clusters. (b) Cell type specific histone mark enrichment within the overlaps. Size and color of the dot indicates significance of the enrichment. Plotted are only enrichments with adjusted p-value < 0.05. (c-e) Annotation of H3K9me3 overlaps with significantly upregulated downregulated H3K27ac regions in one-year-old stunted children. (c) Bar plot showing gene set enrichment results of genes associated with the overlaps. Plotted is the only term with adjusted p-value < 0.05. Source- GO:BP. (d,e) Plots as in (b) showing cell type specific histone mark (d) and DBF (e) enrichment of the overlaps.

# Supplementary tables

Table S1. Preprocessing statistics.

| **dbGaP ID** | **child/mother** | **Number of reads mapped to** | | | **Percent mapped to** | | **Number of hg19 peaks** |
| --- | --- | --- | --- | --- | --- | --- | --- |
|  |  | **total** | **hg19 *** | **dm6 *** | **hg19** | **dm6** |  |
| db2939 | child | 65242578 | 58009506 | 536948 | 88.91 | 0.82 | 21911 |
| db8613 | child | 80047311 | 72313468 | 489027 | 90.34 | 0.61 | 28882 |
| db5113 | child | 95322605 | 86858269 | 1128492 | 91.12 | 1.18 | 23783 |
| db8078 | child | 57913487 | 51020181 | 554225 | 88.10 | 0.96 | 23366 |
| db6596 | child | 64787923 | 57638308 | 910401 | 88.96 | 1.41 | 21147 |
| db2613 | child | 62295875 | 54801957 | 589592 | 87.97 | 0.95 | 22304 |
| db8425 | child | 63293753 | 57541290 | 1109634 | 90.91 | 1.75 | 20293 |
| db5879 | child | 97821145 | 88057861 | 1670967 | 90.02 | 1.71 | 27995 |
| db5597 | child | 52608044 | 47178124 | 762394 | 89.68 | 1.45 | 21471 |
| db3461 | child | 67363529 | 59566683 | 601604 | 88.43 | 0.89 | 24237 |
| db0063 | child | 71032369 | 63234623 | 710232 | 89.02 | 1.00 | 27404 |
| db6561 | child | 56170522 | 50853400 | 455086 | 90.53 | 0.81 | 22708 |
| db0840 | child | 69162107 | 62723121 | 932922 | 90.69 | 1.35 | 21840 |
| db1709 | child | 53902826 | 48773310 | 447933 | 90.48 | 0.83 | 20682 |
| db1120 | child | 78415721 | 69833328 | 500096 | 89.06 | 0.64 | 28708 |
| db4973 | mother | 101305497 | 81880941 | 1597359 | 80.83 | 1.58 | 27469 |
| db2800 | mother | 143271414 | 116312819 | 675620 | 81.18 | 0.47 | 20714 |
| db1172 | mother | 80834859 | 67551277 | 1158579 | 83.57 | 1.43 | 21710 |
| db5558 | mother | 140558171 | 114540924 | 1321667 | 81.49 | 0.94 | 24465 |
| db1967 | mother | 119577348 | 101689539 | 1059786 | 85.04 | 0.89 | 17519 |
| db2875 | mother | 98365763 | 82049321 | 1029596 | 83.41 | 1.05 | 21021 |
| db2368 | mother | 81144570 | 68120605 | 373185 | 83.95 | 0.46 | 18582 |
| db0288 | mother | 81028809 | 64222120 | 1193841 | 79.26 | 1.47 | 21489 |
| db6651 | mother | 112671751 | 94531877 | 1310890 | 83.90 | 1.16 | 11188 |
| db4694 | mother | 113036781 | 89903094 | 1851575 | 79.53 | 1.64 | 26710 |
| db2922 | mother | 124594517 | 103278150 | 2145354 | 82.89 | 1.72 | 20219 |
| db773 | mother | 106652006 | 90193700 | 721800 | 84.57 | 0.68 | 17476 |
| db8636 | mother | 64741297 | 52251876 | 1025601 | 80.71 | 1.58 | 18952 |
| db7005 | mother | 81254146 | 67849919 | 816437 | 83.50 | 1.00 | 18679 |
| * numbers after removal of blacklisted sites | | | | | | | |

Table S2. Sample identifiers and associated measurements.

| **Measurements: mother** | | **height [cm]** | 144 | 151 | 152.5 | 149.4 | 156.6 | 155 | 151.5 | 155 | 151.6 | 150 | 153.1 | 145.7 | 137.4 | 152.1 | 162 | 151 | 139.5 | 151.4 | 146 | 140.9 |
| --- | --- | --- | --- | --- | --- | --- | --- | --- | --- | --- | --- | --- | --- | --- | --- | --- | --- | --- | --- | --- | --- | --- |
| **Info / measurements: child** | **Phenotype** | **1yr** | Mal | Mal | Ctrl | Mal | Ctrl | Ctrl | Ctrl | Ctrl | Ctrl | Mal | Mal | Ctrl | Mal | Ctrl | Ctrl | Ctrl | Mal | Mal | Mal | Mal |
|  |  | **wk18** | Mal | Mal | Ctrl | Mal | Ctrl | Ctrl | Ctrl | Ctrl | Ctrl | Ctrl | Mal | Ctrl | Ctrl | Ctrl | Ctrl | Ctrl | Mal | Ctrl | Mal | Ctrl |
|  |  | **birth** | Ctrl | Ctrl | Ctrl | Mal | Ctrl | Ctrl | Ctrl | Ctrl | Ctrl | Ctrl | Mal | Ctrl | Ctrl | Ctrl | Ctrl | Ctrl | Ctrl | Ctrl | Ctrl | Ctrl |
|  | **ferritin [ng/ml]** | | 59.02 | 12.10 | 79.85 | 17.29 | 16.83 | 29.88 | 24.23 | 15.41 | 44.41 | 23.361 | 50.13 | 21.389 | 45.531 | 30.983 | 55.568 | 17.214 | 44.481 | 34.55 | 21.461 | 39.757 |
|  | **Days of diarr** | **wk18 - birth** | 4 | 5 | 0 | 0 | 16 | 0 | 0 | 4 | 3 | 6 | 11 | 7 | 0 | 0 | 22 | 14 | 27 | 4 | 0 | 1 |
|  | **WAZ** | **1yr** | -2.57 | -3.61 | -0.01 | -3.59 | 1.51 | -0.6 | 1.37 | 0.12 | 1.34 | -1.83 | -2.64 | -1.22 | -2.58 | -0.19 | 0.57 | -0.72 | -3.29 | -1.34 | -2.65 | -1.69 |
|  |  | **wk18** | -1.85 | -2.61 | 0.33 | -3.19 | 1.14 | -1.28 | 1.68 | 0.61 | 1.62 | -0.51 | -1.65 | -0.59 | -1.56 | 0.04 | 1.35 | -0.19 | -2.32 | -1.12 | -2.35 | -1.08 |
|  |  | **birth** | -1.2 | -1.92 | -0.56 | -2.72 | -0.04 | -0.34 | -0.37 | -0.63 | -0.72 | -0.44 | -2.89 | 0.26 | -1.66 | -0.93 | 0.6 | -0.93 | -2.4 | -1.42 | -0.66 | -0.55 |
|  | **∆HAZ** | **1yr - wk18** | -0.97 | -1.43 | -0.37 | -0.24 | -0.05 | 0.2 | 0.64 | -0.36 | 0.24 | -0.96 | 0.53 | -0.88 | -1 | -0.39 | -0.55 | -0.88 | -0.97 | -0.3 | -0.77 | -1.19 |
|  |  | **1yr - birth** | -1.55 | -2.74 | 0.02 | -1.4 | 1.19 | 0.74 | 0.86 | 1.32 | 0.38 | -2.06 | -0.14 | -0.63 | -1.07 | -0.69 | -0.44 | -0.25 | -1.29 | -0.89 | -2.35 | -2.24 |
|  |  | **wk18 - birth** | -0.58 | -1.31 | 0.39 | -1.16 | 1.24 | 0.54 | 0.22 | 1.68 | 0.14 | -1.1 | -0.67 | 0.25 | -0.07 | -0.3 | 0.11 | 0.63 | -0.32 | -0.59 | -1.58 | -1.05 |
|  | **HAZ** | **1yr** | -3.11 | -4.22 | 0.56 | -4.25 | 1.6 | 0.21 | 1.22 | 0.84 | 0.15 | -2.3 | -2.63 | -0.36 | -2.88 | -0.17 | 0.13 | -0.6 | -3.24 | -2.28 | -3.11 | -2.3 |
|  |  | **wk18** | -2.14 | -2.79 | 0.93 | -4.01 | 1.65 | 0.01 | 0.58 | 1.2 | -0.09 | -1.34 | -3.16 | 0.52 | -1.88 | 0.22 | 0.68 | 0.28 | -2.27 | -1.98 | -2.34 | -1.11 |
|  |  | **birth** | -1.56 | -1.48 | 0.54 | -2.85 | 0.41 | -0.53 | 0.36 | -0.48 | -0.23 | -0.24 | -2.49 | 0.27 | -1.81 | 0.52 | 0.57 | -0.35 | -1.95 | -1.39 | -0.76 | -0.06 |
|  | **sex** | | Female | Male | Female | Male | Male | Female | Female | Male | Female | Male | Male | Male | Female | Male | Male | Female | Female | Male | Female | Male |
| **dbGaP ID** | **mother** | | - | db4973 | db2800 | db1172 | db5558 | - | - | db1967 | db2875 | db2368 |  | db0288 | db6651 | - | db4694 | db2922 | db773 | db8636 | - | db7005 |
|  | **child** | | db2939 | db8613 | db5113 | - | db8078 | db6596 | db2613 | db8425 | - | - | db5879 | db5597 | db3461 | db0063 | - | db6561 | db0840 | - | db1709 | db1120 |

# Supplementary text

To determine if the observed global changes to the H3K9me3 profiles with the children’s ΔHAZ (birth:1yr) scores were influenced by previously reported enzymatic cleavage (“clipping”) of histone H3 resulting in loss of H3K9 methylation signal due to N-terminal cleavage [1], we performed Western blotting assessing global H3K9me3 and H3 levels. The results did not suggest histone H3 clipping to be a factor influencing the detectable level of H3K9me3 (Fig. S4). While we do see a secondary band in the H3 Western blots with an apparent molecular weight less than full-length H3, the band is also visible at about the same intensity in the H3K9me3 Western blots. This indicates that the apparent short form of H3 does not results from N-terminal clipping, as Howe & Gamble [1] showed that histone H3 clipping led to loss of the H3K9 methylation signal. The difference in apparent migration of ca. 18 KDa to ca.15 KDa is inconsistent with removal of the first 1-8 amino acids of the H3 tail and more likely this lower molecular weight species results from either a C-terminal truncation of H3 or it could conceivably be a result of cross reactivity with another protein such as H4.

As discussed in more detail in the main body of the manuscript, we report small but statistically robust changes in genomic H3K9me3 with changes in ∆HAZ (birth:1yr) score. We wondered if the global H3K9me3 pattern could be captured by other technologies, such as Western blotting. However, Western blotting is apparently not sensitive enough to reveal these global changes (Fig. S4). At the same time, Western blotting does support the conclusion that large changes in total histone H3, and H3K9me3 or general histone integrity are not driving the observed global changes in profiles of stunted children.

# Supplementary references

1. Howe CG, Gamble M V. Enzymatic cleavage of histone h3: A new consideration when measuring histone modifications in human samples. Clin Epigenetics. 2015 Jan 22;7(1):1–4.
